# Towards predicting intracellular radiofrequency radiation effects

**DOI:** 10.1371/journal.pone.0213286

**Published:** 2019-03-14

**Authors:** Claus Nielsen, Ron Hui, Wing-Yee Lui, Ilia A. Solov’yov

**Affiliations:** 1 Department of Physics, Chemistry and Pharmacy, University of Southern Denmark, Odense M, Denmark; 2 Department of Electrical and Electronic Engineering, The University of Hong Kong, Hong Kong, China; 3 School of Biological Sciences, The University of Hong Kong, Hong Kong, China; Vanderbilt University, UNITED STATES

## Abstract

Recent experiments have reported an effect of weak radiofrequency magnetic fields in the MHz-range on the concentrations of reactive oxygen species (ROS) in living cells. Since the energy that could possibly be deposited by the radiation is orders of magnitude smaller than the energy of molecular thermal motion, it was suggested that the effect was caused by the interaction of RF magnetic fields with transient radical pairs within the cells, affecting the ROS formation rates through the radical pair mechanism. It is, however, at present not entirely clear how to predict RF magnetic field effects at certain field frequency and intensity in nanoscale biomolecular systems. We suggest a possible recipe for interpreting the radiofrequency effects in cells by presenting a general workflow for calculation of the reactive perturbations inside a cell as a function of RF magnetic field strength and frequency. To justify the workflow, we discuss the effects of radiofrequency magnetic fields on generic spin systems to particularly illustrate how the reactive radicals could be affected by specific parameters of the experiment. We finally argue that the suggested workflow can be used to predict effects of radiofrequency magnetic fields on radical pairs in biological cells, which is specially important for wireless recharging technologies where one has to know of any harmful effects that exposure to such radiation might cause.

## Introduction

Weak radiofrequency (RF) magnetic fields in the MHz-range was shown to influence the concentrations of reactive oxygen species (ROS) in living cells [1–4]. Remarkably, the energy that could possibly be deposited by such radiation is orders of magnitude smaller than the energy of molecular thermal motion. A plausible explanation to the observed effect relies on the interaction of RF magnetic fields with transient radicals within the cells, affecting the ROS formation rates through the radical pair mechanism [5–9]. Prediction of the RF magnetic field effects in biomolecular systems is, however, not straightforward, as it relies on multiple interlinked scales ranging from electrons to the whole cell. This gap in our understanding of RF field effects on biological systems is, however, important and needs special attention because wireless charging has already been commercialized in various sectors such as portable consumer electronics [10] and manufacturing facilities [11]. While the World’s first wireless charging standard “Qi” launched by the Wireless Power Consortium focuses on short-range wireless charging which has no danger of human exposure to electromagnetic radiation, mid-range wireless charging (with possible human exposure to electromagnetic radiation) has been suggested recently by a group of companies through the AirFuel Alliance.

A cell contains a vast amount of different components interacting in a myriad of ways, and it is, therefore, no simple task to identify the molecular processes that can be affected by RF magnetic fields. Since radicals could possibly exhibit a sufficiently strong interaction with the weak RF magnetic fields [1, 2, 6, 12–16], the search can be somewhat limited by focusing on molecular intracellular processes involving radicals. Radicals inside a cell may be created in pairs in a coherent state far from thermal equilibrium, and the relaxation pathway towards thermal equilibrium can be altered by weak external magnetic fields [5, 7–9, 17–19]. Due to the high reactivity of radicals, various reaction pathways with radical involvement will normally be available, and external magnetic fields would thus modulate the corresponding reaction probabilities [5–9, 20]. The external magnetic RF fields would, therefore, lead to a difference in the relative amounts of intracellular reaction products that in turn would affect cellular functioning. The effect is expected to be dependent on the strength of the external magnetic fields, as well as their polarization and oscillation frequency.

Examples of radicals interacting with RF magnetic fields are known. For example, in previous studies a radical pair with flavin adenine dinucleotide (FAD) and superoxide ${\text{O}}_{2}^{•-},\left[{\text{FAD}}^{•-}\ldots {\text{O}}_{2}^{•-}\right]$, was suggested to be responsible for an observed effect of RF magnetic fields in human ubilical vein endothelial cells [1, 2], or to be involved in avian magnetoreception [21, 22]. Another example is the cytochrome bc1 complex [23–27], which can be found in the mitochondrial membranes and is responsible for proton transport across the membrane as part of the respiratory chain. A recent study indicated that superoxide may be generated as a side reaction in the cytochrome bc1 complex [24, 25], and it is not unlikely that the ${\text{O}}_{2}^{•-}$ production rate as well as its chance to escape the reaction sites within the protein complex might be affected by RF magnetic fields.

Theoretical description of weak radiofrequency magnetic field effects in biological systems is not new, and several methods that address radical pair spin dynamics have been developed [12, 13, 28–32]. Common to all of these methods is, that they require a specification of a radical pair Hamiltonian, which describes how spins of the unpaired electrons interact with external magnetic fields, the internal magnetic fields of the molecular environment, and with each other. To describe the majority of the radical pair processes, it is often a good approximation to assume the Hamiltonian time-independent [5, 9, 19], which is however not directly possible once an oscillating RF field is present. The burden of dealing with the time-dependent Hamiltonian can, however, be avoided when using the so-called rotating reference frame method [13, 28, 32] which permits rewriting the equations describing radical pair spin dynamics in a time-independent form. Nevertheless, the method has some limitations, as for example it only applies to circularly polarized single-frequency RF magnetic fields. Other methods that do not possess this specific constraint are, for example, based on perturbation theory [29, 30], the so-called Floquet theory [31], or using the *γ*-COMPUTE algorithm which exploits the periodicity of the RF field oscillations [12, 33]; the latter methods are, however, subject to different limitations and approximations.

The present investigation aims to establish a general workflow for studying the impact of RF fields on subcellular compartments. In order to illustrate how one should determine some of the key ingredients of the workflow, the rotating reference frame approach is employed due to its simplicity, making the subject more accessible to researchers without a magnetic resonance or spin dynamics background. It should be emphasized, however, that more sophisticated methods such as the *γ*-COMPUTE algorithm [12, 33] exists and, in principle, allows for a more realistic treatment of intracellular spin systems. Since it is at present not known for any given cell type which radicals would display the largest response to RF magnetic fields, the workflow is justified through examples of generic models of radical pair systems, that are aimed to serve as a foundation for studying RF effects in biological systems of varied complexity, that could be found in real biological environments. The models are essentially used to illuminate how various molecular features of a radical pair affect its response to RF magnetic fields. In particular, we consider how a radical pair system responds to RF magnetic fields for (i) different magnetic interactions within the radical pair described through the so-called hyperfine interactions; (ii) different chemical processes initiated from the radical pair, described through specific reaction rate constants; (iii) different strengths of exchange interaction between the two unpaired electrons. Some of the earlier studies [13, 28, 32] have already addressed these questions for different model systems, but we wish to extend the analysis and discuss the implications of moving from just a simple model system of a radical pair to a more complete description that also involves the cellular scale. Moreover, we aim to deliver a general recipe of how RF magnetic field effects in cells measured in laboratory can be interpreted through computational modelling of the underlying biophysical mechanisms. The performed analysis is aimed at biologists and biochemists interested in RF intracellular effects and seeks to provide a consistent explanation of the underlying physics that could possibly be involved in the observed phenomena.

## Workflow for interpreting RF field effects in cells

**Fig 1:**
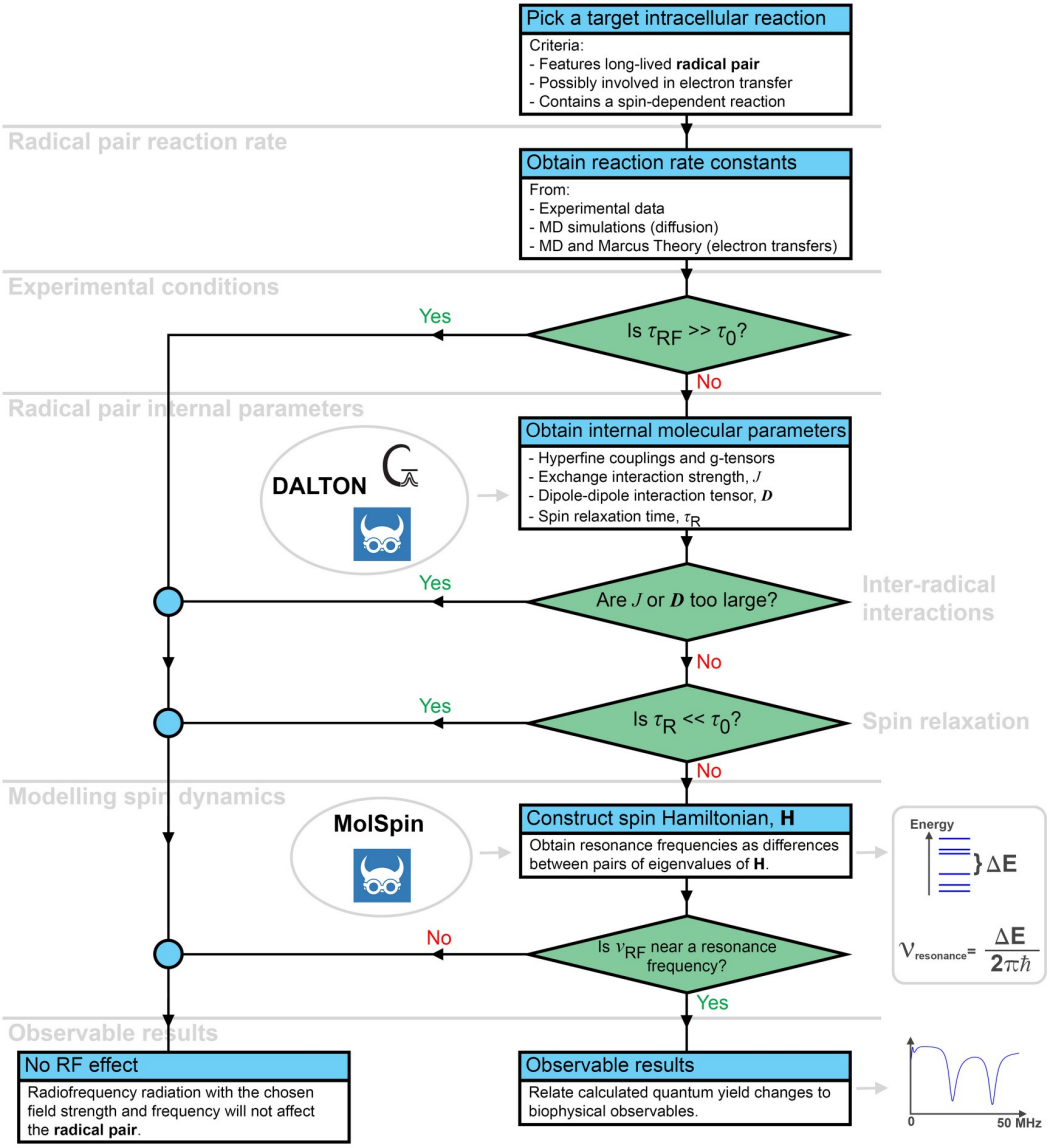
An overview of the workflow to interpret intracellular RF field effects. Three characteristic times are important to expect any possible RF field effects in an intracellular radical pair system: *τ*_0_ is the lifetime of the radical pair; *τ*_*RF*_ is the characteristic time of RF magnetic field action on the radical pair, defined in Eq (2); and *τ*_*R*_ the spin relaxation time. *ν*_*RF*_ is the frequency of the RF field. The steps of the workflow are discussed in the sections of text indicated on the left side of the figure.

Interpretation of RF field effects in real biological systems involves a significant effort, but the workflow outlined in Fig 1 breaks it down into smaller manageable tasks, which are discussed in detail below. All steps in this workflow are essentially relying on a computational approach which should be closely coupled to experiment in terms of defining the variable parameters of RF magnetic fields and of the key observables.

### Radical pair reaction rate constants

Intracellular RF effects depend strongly on the rate constants, associated with the reactions that involve radical pairs. These rate constants determine the fate of the radical pair in terms of possible reaction products and allow estimating the radical pair lifetime. Radical pairs can undergo different processes, where several generic schemes that permit establishing the rate constants rather accurately are reviewed below.

#### Diffusion rates

Some radical pair processes, in particular the backreaction which regenerates the state prior to formation of a non-equilibrium radical pair, can only happen while the radicals are in close proximity. Therefore, some radical pair processes can be suppressed if one of the radicals is able to diffuse away, and thus escape from the other possible radical pair reaction outcomes. The escape rate constant, *k*_*e*_, is related to the binding time *τ*_*b*_ of the two radicals in a radical pair as *k*_*e*_ = 1/*τ*_*b*_. This rate constant can be obtained from molecular dynamics (MD) simulations, where the radical pair and its biological environment have been studied in the state prior to generation of the radical pair, and in a configuration with the radical pair created. Several statistically independent MD simulations of the radical pair state can thus reveal the average binding time, *τ*_*b*_. An example of binding time determination using this approach can be found in an earlier investigation [34].

#### Electron transfer rates

Electron transfer reactions are often associated with radical pair dynamics, as radical pairs are often created or abolished through electron transfer. Charge recombination reactions can often be described by the Marcus theory [35–37], which states that the rate constant of an electron transfer event is given by:

$$\begin{array}{c} k=\frac{2\pi }{\hbar }\frac{|V_{ab}{|}^{2}}{\sqrt{4\pi \lambda k_{B}T}}\exp(-\frac{{\left(\Delta G_{\text{ET}}+\lambda \right)}^{2}}{4\pi \lambda k_{B}T})\;. \end{array}$$ (1)

Here *V*_*ab*_ is the transition matrix element between the two states involved in the charge transfer reaction, *k*_*B*_ the Boltzmann constant, Δ*G*_ET_ is the associated free energy change, and λ is the so-called reorganization energy which describes an additional free energy change linked to the conformational changes caused by the electron transfer [35, 38]. Both Δ*G*_ET_ and λ can be calculated from MD simulations [23, 39], and the transition matrix element can be deduced from quantum chemistry calculations of the radical pair system before and after the electron transfer.

Note that Marcus theory only holds in the adiabatic approximation, where the atomic nuclei can be assumed static relative to the electronic motion. Other methods must be employed for non-adiabatic electron transfers [40].

#### Radical pair lifetime

The lifetime of a radical pair is a crucial characteristic for RF field effects to emerge. The lifetime, *τ*_0_, is defined as the characteristic time where both radicals of the radical pair experience coherent behaviour and can usually be deduced from the fastest spin-independent process associated with the radical pair (for example radical escape diffusion, discussed above); such an estimate delivers a lower bound for the radical pair lifetime, *τ*_0_ = 1/*k* for the rate constant *k*.

If the fastest process in a radical pair is spin-dependent and occurs with a rate constant *k*_*i*_, the initial state of the radical pair becomes important; if this process can happen from the initial state of the radical pair, the corresponding radical pair lifetime is approximated by *τ*_0_ ≈ 1/*k*_*i*_. On the other hand, the radical pair interconversion rate, *k*_mix_, becomes important as the singet-triplet interconversion leads to radical pair conversion into a spin state with a fast decay route characterized by the rate constant *k*_*i*_. For radical pairs, where the singlet-triplet interconversion is limiting the fast spin-dependent process, the radical pair lifetime is approximated by *τ*_0_ ≈ 1/*k*_mix_.

The radical pair lifetime thus often depends on the radical pair interconversion rate, *k*_mix_, which is normally not known upfront since it is mainly determined by the internal parameters of the radical pair such as the hyperfine interactions. As the typical hyperfine interactions are on the order of 1 mT [7, 19, 41], one expects *k*_mix_ to be about $\frac{g\mu_{B}}{2\pi \hbar }\cdot 1\;\text{mT}\approx 30\mu {\text{s}}^{-1}$, which can be used as an order of magnitude guess for the lifetime of a typical radical pair if no other information is available.

### Experimental conditions

One should next consider the experimental conditions which define the external magnetic fields. The strength and direction of any static magnetic field needs to be determined, as well as the strength, frequency, and polarization of the RF magnetic field.

The characteristic interaction time, *τ*_*RF*_, of the RF magnetic fields with the radical pair, can be determined once the strength of the RF magnetic field is known, and is estimated as:

$$\begin{array}{c} \tau_{RF}=\frac{2\pi \hbar }{g\mu_{B}B_{1}}\;. \end{array}$$ (2)

Here *g* is the isotropic Landé *g*-factor which is approximately 2 for organic radicals, *μ*_*B*_ is the Bohr magneton, and *B*_1_ is the RF magnetic field strength. Note that *τ*_*RF*_ depends only on the field strength of the RF magnetic field, *B*_1_, but not on its oscillation frequency or polarization. If the interaction time is much larger than the lifetime of the radical pair, *τ*_*RF*_ ≫ *τ*_0_, the RF magnetic field will not have sufficient time to interact with the radical pair before it decays and, therefore, any effect of the RF magnetic field on the radical pair can be readily excluded, as also schematically depicted in Fig 1.

### Radical pair internal parameters

The internal parameters describe the interactions within each radical pair, and knowledge of those can sometimes be enough to exclude the possibility of RF magnetic field effects on the biochemical system, as illustrated in Fig 1. The most important parameters describing internal interactions in the radical pairs are the so-called hyperfine couplings, describing the interaction between the unpaired electrons of the radical pairs and any nearby magnetic atomic nuclei, which are present in most radicals. The hyperfine interaction is often decomposed into the spin-dipole interaction, which provides the anisotropic part of the hyperfine interaction, and the Fermi Contact term which is isotropic. Most standard quantum chemistry software packages like Gaussian [42] or DALTON [43] are able to calculate these two components of the hyperfine interactions.

#### Inter-radical interactions

The two unpaired electrons of the radical pair may interact with each other through the exchange interaction [44–46], **H**_*ex*_, or magnetic dipole-dipole interaction [45, 46], **H**_*dd*_, which have the following generic form:

$$\begin{array}{cc} H_{ex} & =-2J\left(r\right)\;S_{1}\cdot S_{2}, \end{array}$$ (3)

$$\begin{array}{cc} H_{dd} & =\frac{g_{1}g_{2}\mu_{0}\mu_{B}^{2}}{4\pi }(\frac{3\left(S_{1}\cdot r\right)\left(r\cdot S_{2}\right)}{{\left|r\right|}^{5}}-\frac{S_{1}\cdot S_{2}}{{\left|r\right|}^{3}})=S_{1}\cdot D\left(r\right)\cdot S_{2}\;. \end{array}$$ (4)

Here *J*(**r**) is the strength of the exchange interaction, **D**(**r**) the magnetic dipole-dipole interaction tensor, **r** the vector going from one radical to the other, **S**_1_ and **S**_2_ are the electron spin operators, and *g*_1_ and *g*_2_ are the *g*-factors of the two radicals. Since the unpaired electrons of the two radicals are not located at a specific point, integration over their wavefunctions is necessary in order to obtain accurate *J* and **D** parameters. The effect of *J* and **D** is mainly to suppress singlet-triplet interconversion in the radical pairs, which in turn can render the radical pairs immune to RF magnetic field effects, as highlighted in Fig 1. Both interactions strongly depend on the interradical distance *r* and, therefore, significant variations of *J* and **D** may occur for unconstrained radical pairs. Variation of *J* and **D** is furthermore a source of spin relaxation.

#### Spin relaxation

Most of the interactions in a radical pair are not static, but depend on time due to the atomic thermal motions. Instead of trying to solve the spin dynamics equations with time-dependent internal interactions, the associated effects can be accounted for through the Redfield theory [18, 45, 47, 48], as was for example done earlier [18, 48]. Accurate accounting for spin relaxation can be complicated, and sometimes it is sufficient to estimate the spin relaxation time, *τ*_*R*_, which describes the time at which the spin system will reach thermal equilibrium due to relaxation effects. If the relaxation time is much shorter than the expected lifetime of the radical pair, i.e. *τ*_*R*_ ≪ *τ*_0_, the radical pair will reach thermal equilibrium quickly, and will not be able to respond to RF magnetic fields as indicated in Fig 1. If, on the other hand, the relaxation time is much longer than the lifetime of the radical pair, relaxation becomes irrelevant and can be ignored.

### Modelling spin dynamics

Once the magnetic and kinetic properties of a radical pair are established it is possible to describe the underlying spin dynamics. A simple initial analysis can provide important insights into the spin system: the eigenvalues of the radical pair spin Hamiltonian, in the absence of the RF magnetic field can be obtained. The difference between any two eigenvalues, Δ*E*_*ij*_, corresponds to a resonance frequency in the spin system and, therefore, the calculated eigenvalues provide a complete spectrum of possible resonance frequencies in the studied radical pair, see Fig 1. Not all of the resonance frequencies, however, will affect the quantum yield of the radical pair reaction. This simple analysis can thus immediately exclude any RF magnetic field effect of certain frequencies, that are significantly different from the deduced resonance frequencies, since they would not be able to affect the quantum yield at all. For organic radicals one typically expects multiple resonance frequencies within the 1-50 MHz range due to the hyperfine interactions of the radicals.

For the calculation of the quantum yield change as a function of RF frequency, one can for example use the MolSpin [49] software package, either directly or through the intuitive interface in VIKING [50], which is easy to use and well suited for such calculations.

### Observable results

The quantum yields delivered from the spin dynamical calculations can finally be used to qualitatively judge about the relative amount of reaction products obtained from the various reaction pathways associated with the radical pair. Comparing the quantum yields with and without the presence of a RF field, one can thus conclude on the impact of RF magnetic fields on the relative amount of reaction products inside a cell and ultimately suggest if any noticable effects (cell lifetime, growth rate, etc.) are expected due to the presence of a certain radical pair and its involvement in one of the aforementioned processes.

## Employing the workflow for generic model systems

**Fig 2:**
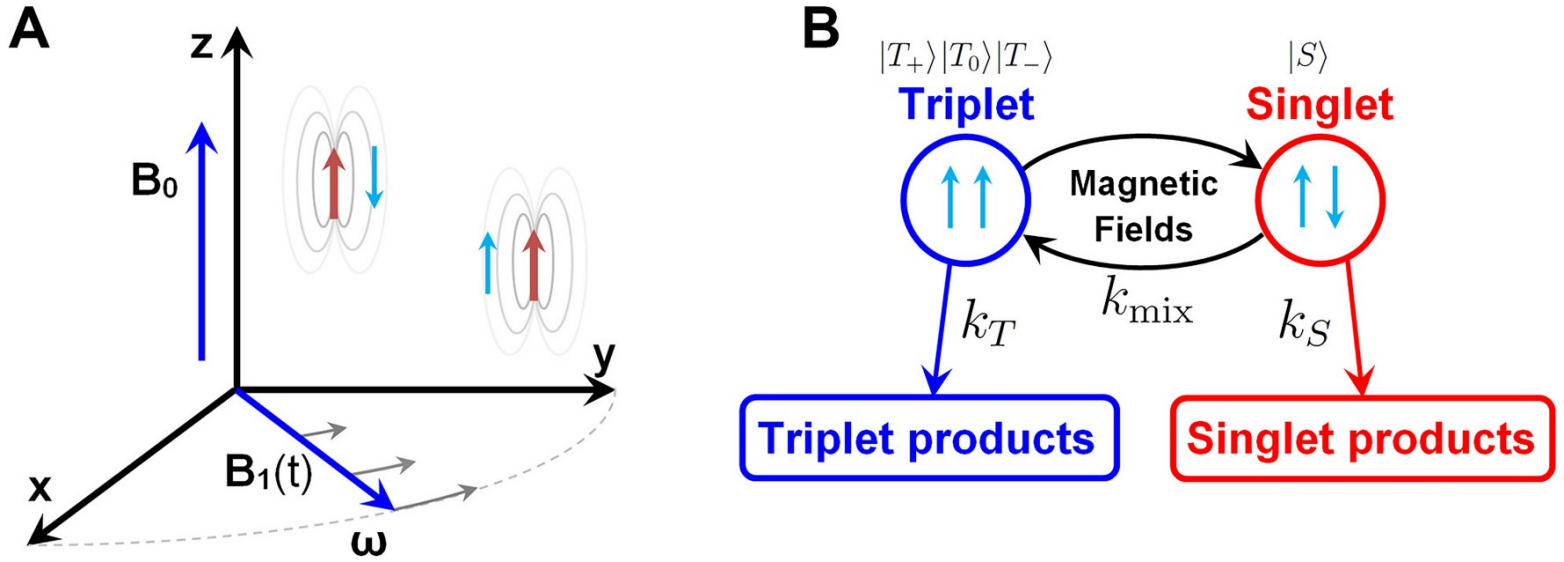
Overview of the simple radical pair model. **A**: The static magnetic field **B**_0_ points along the *z*-axis, and the RF field **B**_1_(*t*) acts in the *xy*-plane. The unpaired electrons are each localized around the magnetic nuclei. **B**: Magnetic interactions in the system induces singlet-triplet mixing within the radical pair with a characteristic rate constant *k*_mix_. Chemical reactions can occur with rate constants *k*_*S*_ and *k*_*T*_ from radical pairs in the singlet and triplet state, respectively, limiting the lifetime *τ*_0_ of the radical pair.

To employ the generic workflow in Fig 1, it is illuminating to consider simple generic radical pair models. An example of a simple radical pair model is illustrated in Fig 2A, consisting of two magnetic nuclei (red arrows) and two unpaired electrons (blue arrows), such that each radical has a single magnetic nucleus. The unpaired electrons in the radical pair posses a property called spin [44], which permits them to be affected by external magnetic fields, such as RF magnetic fields. The external magnetic fields, as well as other magnetic interactions of the unpaired electronic spins, result in the singlet-triplet mixing of the radical pair, which is an interconversion between two different types of quantum mechanical states that the spins of the unpaired electrons can reside in, called singlet and triplet states, and occuring with a characteristic rate constant *k*_mix_. More details about the quantum states of a radical pair can be found in the Supporting Information (SI). The significance of the singlet and triplet states is illustrated in Fig 2B, and note also the presence of two spin-dependent processes occuring with rate constants *k*_*S*_ and *k*_*T*_ from the singlet and triplet state, respectively. These processes could, for example, be electron transfer processes, and will (possibly together with *k*_mix_) determine the lifetime of the radical pair, *τ*_0_. The oversimplified radical pair model system in Fig 2 is expected to be more complex in reality and include more nuclei [15, 48, 51], and, therefore, more local magnetic interactions, that will add a specific signature to how a radical pair will respond to external magnetic fields. A more complex model including more magnetic nuclei is, therefore, also considered below. The minimal model, however, is supposed to illuminate in an intuitive fashion the principal effects that are expected to arise in a radical pair system, once it is subject to RF magnetic fields.

### Measuring RF field effects in an experiment

The RF magnetic fields could affect cellular dynamics in various ways, such as for example the cell growth or cell death rate. The RF magnetic field influence depends on the underlying subcellular structures and chemical processes on the molecular scale, some of which are involved in radical production. The produced radicals can appear crucial for the functioning of molecular systems [6], or become unfortunate side products as for example the superoxide produced in the bc1 complex [24, 25]. Radicals, and radical pairs in particular, can be created during vital cellular processes, and the spin dynamics of these radicals may determine the outcome of chemical processes in which they participate. In the cytochrome bc1 complex, for example, the radical spin dynamics could possibly affect the probability for ${\text{O}}_{2}^{•-}$ to leave the complex without recombining back to O_2_, thus changing the superoxide concentration within the cell and disturbing the normal operation of the bc1 complex, which rely on intrinsic electron transfers.

To make the proposed approach less abstract, we would like to consider a possible experimental setup, where RF field effects can be observed, and the suggested workflow would turn out to be useful in interpreting observables. For the sake of simplicity it will be assumed that in a staged experiment every cell contains the same number of radical pairs that is affected by RF magnetic fields. Introducing a varying number of radical pairs in different cells does not change the outcomes of the modelling conceptually, and should be accounted for by averaging over an ensemble of cells. Each radical pair leads to different chemical reactions that are initiated from the singlet and triplet states, leading to the so-called singlet and triplet yields; such singlet and triplet yields could for example be superoxide recombining to form O_2_ and superoxide escaping the bc1 complex, respectively.

**Fig 3:**
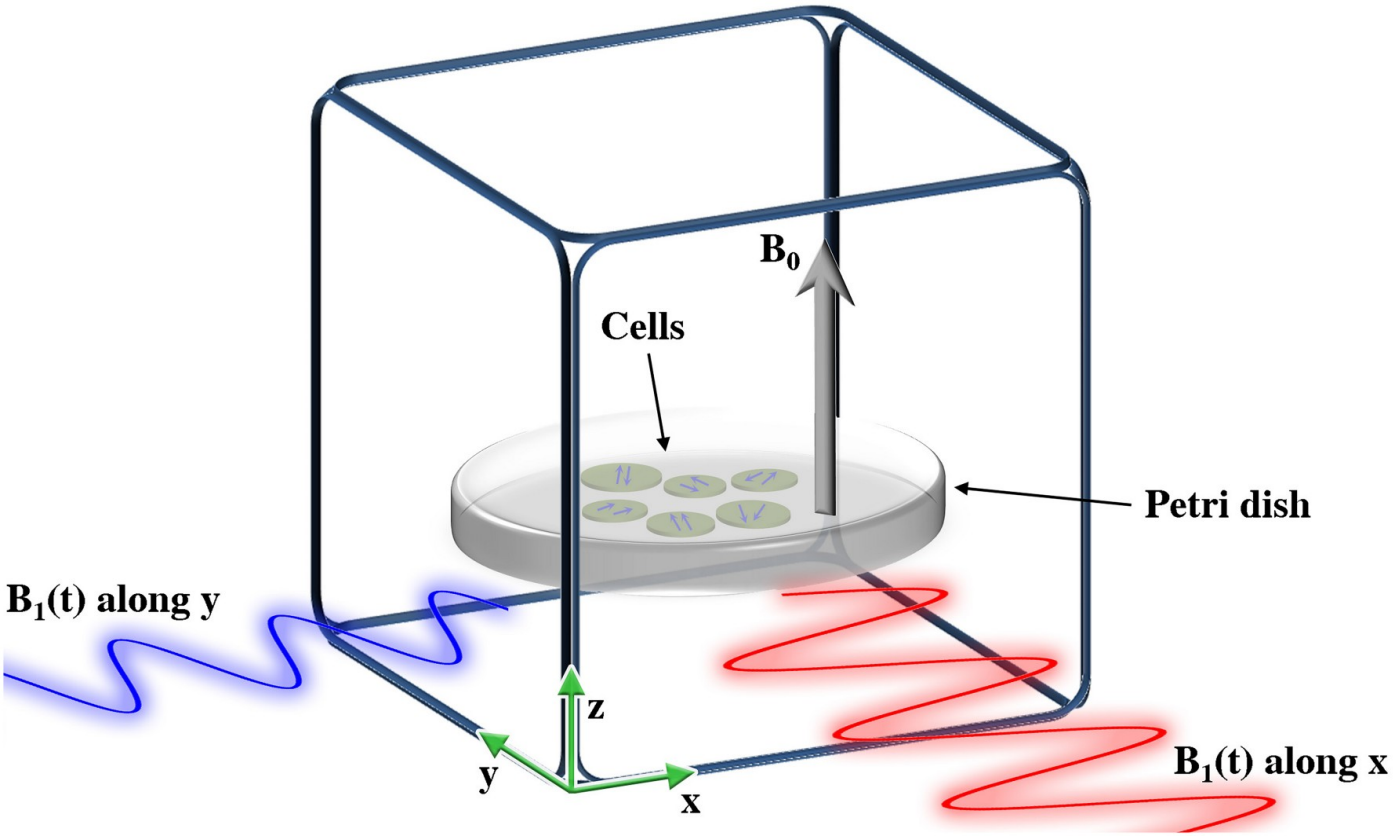
The laboratory coordinate frame. An artistic representation of a Petri dish with an ensemble of cells, and a magnetic field, *B*_0_, in the direction perpendicular to the plane of the Petri dish. The Petri dish is surrounded by rectangular coils that can produce an oscillating magnetic field *B*_1_ along any of the three axes in the laboratory frame. Thus an RF magnetic field rotating in the plane of the Petri dish could be produced, cf. Fig 2A. The orientation of each cells, and hence also of any radical inside the cells, is random, which dictates that any orientational dependent property of the radical pairs should be averaged over all the possible radical orientations.

To quantify the action of the magnetic field, it is important to define a coordinate frame used to describe the radical pairs as illustrated in Fig 2A. The most intuitive choice of the coordinate frame is the laboratory frame, illustrated in Fig 3; assuming we have an experimental setup where some cells are put in a Petri dish, and subject to the static and oscillating magnetic fields *B*_0_ and *B*_1_, respectively. The static homogeneous magnetic field could then be applied in a direction perpendicular to the Petri dish, and thereby determine the *z*-axis; the plane of the Petri dish would then correspond to the *xy*-plane.

The radical pairs are assumed to be floating freely inside the cells, such that they would feature all possible orientations of the corresponding magnetic moments. Changing the orientation of the cell does not change the internal interactions within the cell, but it does have an effect on interactions with external entities, such as the static external magnetic field, as well as the RF magnetic fields. The experiment is thus expected to be performed on an ensemble of radical pairs, which include all possible orientations of the radical pairs relatively to the external magnetic fields. Averaging over all radical pair orientations is straightforward when all internal interactions in the radical pair, such as e.g. the internal magnetic hyperfine interactions, are isotropic, since in that case the spin dynamics of the radical pair become independent of the external magnetic field direction. Normally, however, hyperfine interactions are anisotropic, but unless the radicals are constrained in some way, rotational diffusion of the radicals will smear away the anisotropies. This happens if the characteristic time for rotational diffusion, the rotational correlation time *τ*_rot_, is significantly smaller than the characteristic time scale for the spin dynamics, i.e. the time scale for the singlet-triplet mixing 1/*k*_mix_ = *τ*_mix_, see Fig 2B. Typical rotational correlation times for free organic radicals are on the order of *τ*_rot_ = 1 ps [45], while the time scale of the processes associated with spin dynamics are of ns to *μ*s duration, as shown below. The huge difference in the characteristic values of *τ*_rot_ and *τ*_mix_ suggests that all the hyperfine interactions in a typical unconstrained intracellular radical pair can be considered as isotropic to a good approximation [7, 8]. The situation is more complicated for constrained radical pairs, where one has to take the anisotropy into account.

## Model details

In order to characterize an ensemble of radical pairs, one should first consider how a single radical pair is described. The results of such a description are the so-called quantum yields, which determine the probability for reaction pathways for the single radical pair. Since most of the radical pairs in an experiment are expected to be similarly prepared, they are assumed to be spawned in the same quantum state, and experience the same internal interactions as the molecular structures of the radical pairs are thought identical. The calculated probabilities for the various reaction products in a single radical pair could then be generalized for every radical pair in the experimental system. There are two potential problems with this approach: (i) the radical pairs in the experimental setup have different orientation relative to external static and RF magnetic fields, and (ii) the inherent randomness of the thermal motion present in the radicals will lead to different molecular motions for each of the radical pairs in the ensemble. The first problem is solved by averaging the yield obtained for a single radical pair over all possible orientations of this radical pair. The second problem can be handled through the Redfield theory [18, 45, 47, 48], which has been developed to take spin relaxation into account.

For the description of a single radical pair, one must first account for all the interactions with the magnetic nuclei within the radicals. Such intrinsic magnetic fields of the nuclei are described by the hyperfine interactions which in the present study are assumed to be isotropic, following the arguments above. The strength of the isotropic hyperfine interaction between an unpaired electron of a radical and a magnetic nucleus is characterized by the hyperfine coupling. Since the simple model radical pair shown in Fig 2A has two magnetic nuclei, the hyperfine coupling *a*_1_ is a parameter that permits altering the magnetic properties of the first nucleus, while the hyperfine coupling of the other nucleus, *a*_2_ = 1 mT, is kept constant in all calculations. The values for the hyperfine coupling are chosen such that they represent the common order of magnitude for hyperfine couplings for magnetic nuclei [7, 19, 41], and the hyperfine interactions are assumed to be isotropic due to rotational diffusion of the radicals as explained above. Each nucleus in the studied simple radical pair, Fig 2A, is assumed to have a spin quantum number of 1/2, and the Hamiltonian describing the hyperfine interactions of the unpaired electronic spins with the nuclei reads as:

$$\begin{array}{c} H_{HF}=g\mu_{B}(a_{1}S_{1}\cdot I_{1}+a_{2}S_{2}\cdot I_{2})\;. \end{array}$$ (5)

Here **S**_1_ and **S**_2_ are the electronic spin operators for the two unpaired electrons, and likewise **I**_1_ and **I**_2_ are the two nuclear spin operators. The product **S**_1_ ⋅ **S**_2_ is defined as **S**_1_ ⋅ **I**_1_ = **S**_1*x*_ **I**_1*x*_ + **S**_1*y*_ **I**_1*y*_ + **S**_1*z*_ **I**_1*z*_ and similarly for **S**_2_ ⋅ **I**_2_ [44, 52, 53]. The radical pair system is assumed to be exposed to the static magnetic field **B**_0_, which points along the *z*-axis in the laboratory coordinate frame, see Figs 2 and 3, as well as the time-dependent magnetic RF field **B**_1_(*t*). The interaction between the electronic spins and both of these magnetic fields is described by the Zeeman Hamiltonian, as

$$\begin{array}{c} H_{Z}\left(t,\Omega \right)=g\mu_{B}\left(R\left(\Omega \right)S_{1}+R\left(\Omega \right)S_{2}\right)\cdot [B_{0}\;\hat{z}+B_{1}\;\left(\hat{x}\cos\left(\omega t+\phi \right)+\hat{y}\sin\left(\omega t+\phi \right)\right)], \end{array}$$ (6)

where $\hat{x}$, $\hat{y}$ and $\hat{z}$ are the unit vectors pointing along the *x*-, *y*- and *z*-axes, respectively, *ω* is the angular frequency of the RF magnetic field, *B*_1_ is the RF magnetic field strength, and *ϕ* is the phase of the RF magnetic field. **R**(**Ω**) is a standard 3 × 3 rotation matrix (defined in the SI), where Ω denotes the orientation of the radical pair relative to the laboratory reference frame illustrated in Fig 3. The rotation matrix is necessary for all interactions between internal entities, such as the electronic spins or magnetic nuclei, and external entities, such as the static and RF magnetic fields, since **R**(Ω) is the matrix that transforms the spin operators from the molecular reference frame to the laboratory reference frame; this ensures that the calculation results are independent of the choice of the reference frame. Note that the rotation matrix was not necessary in Eq (5) since only internal entities were involved there.

The nuclear Zeeman term describing the interaction between the external magnetic field and the nuclear spins is neglected in Eq (6), as it is 2-3 orders of magnitude smaller than the electronic Zeeman term due to the magnetic moment of an electron and nuclei being inversely proportional to their respective masses.

The Zeeman Hamiltonian in Eq (6) may look rather complicated, and it is therefore instructive to write the static and RF contributions explicitly:

HZ,B0(t,Ω)=gμBB0(Rzx(S1x+S2x)+Rzy(S1y+S2y)+Rzz(S1z+S2z)),HZ,B1(t,Ω)=gμBB1[(Rxx(S1x+S2x)+Rxy(S1y+S2y)+Rxz(S1z+S2z))cos(ωt+ϕ)=+(Ryx(S1x+S2x)+Ryy(S1y+S2y)+Ryz(S1z+S2z))sin(ωt+ϕ)]. (7)

Here *R*_*ij*_ are the components of the rotation matrix, **R**(Ω), where the orientation Ω has been omitted for clarity. In the special case where the molecular reference frame is the same as the laboratory reference frame, the rotation matrix becomes the identity operator, where *R*_*xx*_ = *R*_*yy*_ = *R*_*zz*_ = 1, while all other components are zero. In this case Eq (7) reads as:

$$\begin{array}{cc} H_{Z,B_{0}}\left(t,\Omega \right) & =g\mu_{B}B_{0}\;\left(S_{1z}+S_{2z}\right),\\ H_{Z,B_{1}}\left(t,\Omega \right) & =g\mu_{B}B_{1}[\left(S_{1x}+S_{2x}\right)\cos\left(\omega t+\phi \right)+\left(S_{1y}+S_{2y}\right)\sin\left(\omega t+\phi \right)]\;. \end{array}$$ (8)

This simple case only holds when all internal interactions are isotropic, as discussed below, while Eqs (6) and (7) are more general.

The two unpaired electrons in the radical pair are assumed to have oppositely aligned spins initially, i.e. assume the radicals to be created in the coherent singlet state, denoted as |*S*〉. This singlet state is not an eigenstate of the hyperfine Hamiltonian, and is a superposition of the different quantum states which evolve differently over time. Thus the radical pair will alternate between the singlet state |*S*〉 and the three triplet states |*T*_0_〉, |*T*_+_〉 and |*T*_−_〉 with a characteristic rate *k*_mix_, see Fig 2B, and this dynamics could be affected by RF magnetic fields once the field frequency matches the energy difference between any two quantum states of the radical pair, see Fig 1. A more detailed explanation of singlet and triplet states, and why they are mixed by the Hamiltonian operator, is given in the SI.

In addition to the singlet-triplet mixing of the radical pair, the radicals are expected to participate in chemical reactions within the cell. These reactions depend on the spin state of the radical pair, and it is, therefore, generically assumed that different reactions happen from the singlet and the triplet states [54], leading to the distinct reaction rate constants *k*_*S*_ and *k*_*T*_, respectively, as illustrated in Fig 1B.

The dynamics of the radical pair can be described in terms of the density operator, *ρ*(*t*, Ω), and its time evolution is described by the Liouville-von Neumann equation [9, 15, 52, 55–58]:

$$\begin{array}{c} \frac{\partial \rho (t,\Omega )}{\partial t}=-\frac{i}{\hbar }{\left[H\left(t,\Omega \right),\rho \left(t,\Omega \right)\right]}_{-}-\frac{k_{S}}{2}{\left[P_{S},\rho \left(t,\Omega \right)\right]}_{+}-\frac{k_{T}}{2}{\left[P_{T},\rho \left(t,\Omega \right)\right]}_{+}\;. \end{array}$$ (9)

Here [*A*, *B*]_∓_ = *AB* ∓ *BA* denote the commutator and anti-commutator, while $P_{S}=\frac{1}{4}1-S_{1}\cdot S_{2}$ and $P_{T}=\frac{3}{4}1+S_{1}\cdot S_{2}$ are the singlet and triplet projection operators, respectively. The last two terms in Eq (9) describe the chemical reactions [20], while the rest of Eq (9) is a reformulation of the Schrödinger equation in the density operator formalism [9, 15, 45, 48, 52, 55, 56]. All details about the dynamics of the system and the RF magnetic field are stored in the Hamiltonian operator, **H**(*t*, Ω) = **H**_*HF*_ + **H**_*Z*_(*t*, Ω), and it is the hyperfine interaction **H**_*HF*_, Eq (5), that is responsible for the singlet-triplet mixing between the different spin states of the radical pair. The static and RF magnetic fields in turn affect the mixing rates of the spin states. Note that solution of Eq (9) in its general form becomes difficult due to the time-dependent Hamiltonian, **H**(*t*, Ω), and special methods have been developed to handle such cases—the simplest of these is probably the rotating reference frame approach [13, 28, 32].

The probability for the radical pair to reside in a specific spin state determines the corresponding quantum yield of an associated reaction, and read as [59, 60]:

$$\begin{array}{c} \phi_{S}\left(t,\Omega \right)=\mathrm{Tr}\left[P_{S}\rho \left(t,\Omega \right)\right]\;. \end{array}$$ (10)

Specifically, this is the probability of finding a radical pair in the singlet state at the time instance *t* [9, 15, 48, 52, 56], and *ϕ*_*T*_ = Tr[**P**_*T*_ *ρ*(*t*, Ω)] defines the probability to observe the triplet state. The total singlet quantum yield, i.e. the probability of forming the singlet products from a radical pair, see Fig 2B, is then given by:

$$\begin{array}{c} \Phi_{S}\left(\Omega \right)=\int_{0}^{\infty }k_{S}\phi_{S}\;dt\;. \end{array}$$ (11)

Likewise the total triplet quantum yield, Φ_*T*_(Ω), can be defined in terms of the time integral of the triplet fraction, i.e. $\Phi_{T}\left(\Omega \right)=\int_{0}^{\infty }k_{T}\phi_{T}\;dt$. The quantum yields Φ_*S*_(Ω) and Φ_*T*_(Ω) thus provide the probability that the radical pair ends up as the singlet or the triplet product, respectively.

Once the quantum yields for a single radical pair are obtained, one should consider the ensemble of radical pairs. Since the radical pairs in the ensemble will have different orientations, Ω, one should average the quantum yields over all possible orientations in order to obtain the ensemble average:

$$\begin{array}{c} \left\langle \Phi_{S}\right\rangle =\frac{1}{8\pi^{2}}\int \int_{0}^{\infty }k_{S}\phi_{S}\;dt\;d\Omega \;. \end{array}$$ (12)

Here the prefactor of $\frac{1}{8\pi^{2}}$ is a normalization constant. A reference frame can be described by three Euler angles, *α*, *β*, and *γ*. The ensemble average in Eq (12) may therefore be be written as:

$$\begin{array}{c} \left\langle \Phi_{S}\right\rangle =\frac{1}{8\pi^{2}}\int_{0}^{2\pi }\int_{0}^{2\pi }\int_{0}^{\pi }\int_{0}^{\infty }k_{S}\phi_{S}\;dt\;\sin\left(\beta \right)d\beta \;d\alpha \;d\gamma \;. \end{array}$$ (13)

If the density operator, *ρ*(*t*, Ω) in Eq (10), is independent of the orientation of the radicals due to e.g. rotational diffusion, Eq (13) simplifies to:

$$\begin{array}{c} \left\langle \Phi_{S}\right\rangle =\int_{0}^{\infty }k_{S}\phi_{S}\;dt\;. \end{array}$$ (14)

Note, however, that this simplification is not possible for anisoptropic hyperfine interactions (i.e. if the radicals are constrained), or once other anisotropic interactions such as the magnetic dipole-dipole interaction are present, which may be significant when the radicals are located close to each other. The generic model systems studied here only have isotropic internal interactions and so Eq (14) holds.

The ensemble average of the quantum yield determines the fraction of radical pairs reacting through a particular reaction pathway; in the generic model system described in Fig 2B it describes the fraction of radical pairs that end up as singlet and triplet products, respectively. If *N*_RPs_ denotes the number of radical pairs within a cell, and *N*_cells_ the total number of cells in an experiment, the total yields can, therefore, be defined as:

$$\begin{array}{c} \bar{\left\langle \Phi_{S}\right\rangle }=N\left\langle \Phi_{S}\right\rangle ,\;\bar{\left\langle \Phi_{T}\right\rangle }=N\left\langle \Phi_{T}\right\rangle ,\;N=N_{\text{RPs}}N_{\text{cells}}\;. \end{array}$$ (15)

Thus the average total yields $\bar{\langle \Phi_{S}\rangle }$ and $\bar{\langle \Phi_{T}\rangle }$, or the ratio $\bar{\langle \Phi_{S}\rangle }/\bar{\langle \Phi_{T}\rangle }$, are the quantities that can be measured experimentally [59, 60] if intracellular reactions that rely on radical pair dynamics are somewhat known. It is thus only the RF field effects that causes a change in these average total yields, and hence the average quantum yields, that are becoming noticeable on the cellular scale. Since the exact number of radicals N is normally not known, it is more common to instead consider the fractions:

$$\begin{array}{c} \frac{\bar{\left\langle \Phi_{S}\right\rangle }}{\bar{\left\langle \Phi_{S}\right\rangle }+\bar{\left\langle \Phi_{T}\right\rangle }}=\left\langle \Phi_{S}\right\rangle ,\;\frac{\bar{\left\langle \Phi_{T}\right\rangle }}{\bar{\left\langle \Phi_{S}\right\rangle }+\bar{\left\langle \Phi_{T}\right\rangle }}=\left\langle \Phi_{T}\right\rangle , \end{array}$$ (16)

which can be compared with experimentally measured fractions of the various reaction products.

## Results of model calculations

### Quantifying radical pair response to RF fields

**Fig 4:**
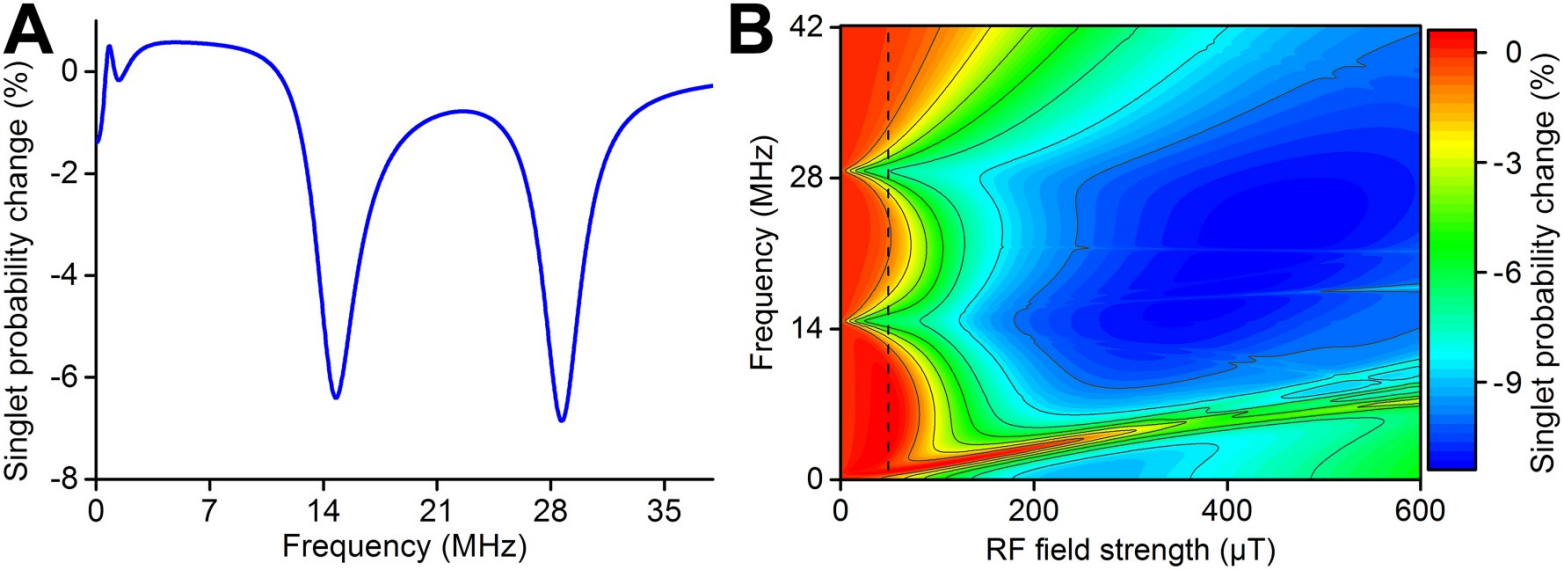
Possible singlet product probability change in the radical pair. **A**: An action spectrum showing the singlet product probability change, Eq (17), as a function of RF magnetic field frequency, *ω*, for a specific field strength, *B*_1_ = 50 *μ*T, as defined in Eq (6). Note that the yield change is non-zero at zero frequency, since the RF magnetic field corresponds to a static field in this case, i.e. setting *ω* = 0 in Eq (6). **B**: The singlet product probability change, Eq (17), as a function of both *ω* and *B*_1_. Note that **A** is the vertical slice of **B** with *B*_1_ = 50 *μ*T, as indicated by the dashed line in **B**. All calculations assume *k*_*S*_ = *k*_*T*_ = 10^6^ s^−1^, *B*_0_ = 50 *μ*T and *a*_1_ = 0.5 mT.

Evaluating the probability of forming the singlet products of the radical pair reaction with and without the presence of a RF magnetic field, and by subtracting one from another, one obtains an action spectrum as shown in Fig 4A. This spectrum describes specifically the change of the ensemble averaged singlet quantum yield in the model radical pair system, and is mathematically defined as:

$$\begin{array}{c} \Delta \left\langle \Phi_{S}\right\rangle =\left\langle \tilde{\Phi_{S}}\right\rangle -{\left\langle \Phi_{S}\right\rangle }_{0}\;. \end{array}$$ (17)

Here $\langle \tilde{\Phi_{S}}\rangle$ is the average of singlet products describing the ensemble of radical pairs in the presence of an RF magnetic field, while 〈Φ_*S*_〉_0_ is the same quantity without the RF contribution, i.e. corresponding to *B*_1_ = 0. Thus $\langle \tilde{\Phi_{S}}\rangle$ and 〈Φ_*S*_〉_0_ corresponds to two different experiments: one with a RF magnetic field present and another experiment without, respectively. The difference between the average singlet product yields in the two experiments, Δ〈Φ_*S*_〉, allows then to quantify the effect of the RF magnetic field. Note that although the definition of Δ〈Φ_*S*_〉 in Eq (17) makes the singlet product probability difference a function of both *B*_1_ and *ω*, other parameters such as reaction rate constants and hyperfine couplings still influence the radical pair dynamics, and, therefore, the reaction yield change. The singlet product probability change, Δ〈Φ_*S*_〉 for this model system, reflects the concentration changes in a chemical species as a result of the RF magnetic field, thus the calculation results are linked to physical observables. It is then possible to obtain action spectra for different combinations of *B*_1_ and *ω*, as done in Fig 4B for the studied model radical pair system with *k*_*S*_ = *k*_*T*_ = 10^6^ s^−1^, *B*_0_ = 50 *μ*T and *a*_1_ = 0.5 mT, see Fig 2. Note that clear peculiarities are seen in Fig 4B for very weak RF magnetic fields of *B*_1_ < 100 *μ*T, while these features disappear as *B*_1_ approaches the strength of the hyperfine interactions in the radical pair which in the considered example are 500-1000 *μ*T. This behaviour is expected to arise because the RF magnetic field induces transitions between spin states at a rate comparable with the rate for coherent mixing of singlet and triplet states governed by the hyperfine interactions, thereby interfering strongly with the coherent spin dynamics in the radical pair.

### Influence of the internal magnetic fields

**Fig 5:**
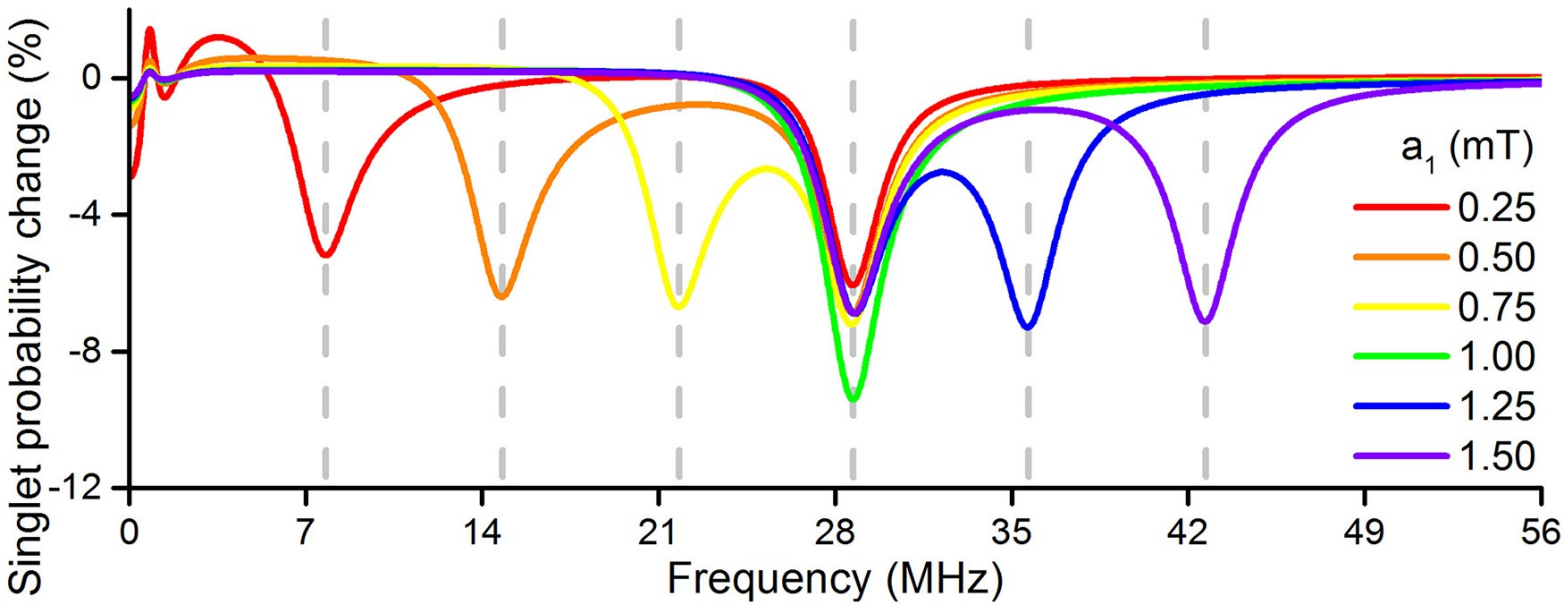
Hyperfine interactions impact the singlet product probability change in the studied model reaction. The strength of the isotropic hyperfine coupling constant *a*_1_ is modified here. Pronounced changes in the singlet probability are always seen in the low-frequency limit, and for $\omega \approx a_{1}\cdot 28\frac{\text{MHz}}{\text{mT}}+0.7\;\text{MHz}$. The fixed hyperfine coupling, **a**_2_, gives rise to a singlet probability change around 28.7 MHz and enhances the singlet probability change at 28.7 MHz when *a*_1_ = 1.00 mT. *k*_*S*_ = *k*_*T*_ = 10^6^ s^−1^, *B*_0_ = 50 *μ*T and *B*_1_ = 50 *μ*T are assumed in all calculations.

The singlet and triplet states of the radical pair are the so-called eigenstates of the Zeeman interaction with a homogeneous magnetic field along the *z*-axis, and eigenstates of the Hamiltonian do not mix over time. It is, therefore, necessary to include the hyperfine interactions of at least one magnetic nucleus in order to enable any possibility for singlet-triplet mixing, since singlet and triplet states will no longer be eigenstates of the total Hamiltonian in that case. In practice this means that radical pairs without any magnetic nuclei, and therefore no hyperfine interactions, such as a pair of superoxide radicals, would not have any singlet-triplet mixing (unless it is introduced by other means, such as *g*-tensor anisotropy) and, therefore, no RF magnetic field effects would be possible. A single hyperfine coupling on at least one of the two radicals is enough to enable singlet-triplet mixing. The reason that hyperfine interactions are necessary for state mixing is due to the conservation of angular momentum: the singlet and triplet states have different angular momenta by definition, and a conversion between singlet and triplet states can, therefore, only happen if an external angular momentum, such as a nuclear spin, is changed simulatneously, in order to conserve the total angular momentum. RF magnetic fields can also be described in terms of photons, and since photons carry angular momentum, interactions with photons can cause transitions between spin states as well. In the model radical pair system investigated here, each radical has a single magnetic nucleus, and the effect of changing the internal magnetic interactions within the radical pair, i.e. the hyperfine interaction strength of one of these nuclei, through changing *a*_1_, is explored in Fig 5. The obtained change in the singlet product probability can be understood by rewriting the hyperfine interaction for the first radical, as:

$$\begin{array}{c} H_{HF}^{\left(1\right)}=g\mu_{B}a_{1}S_{1}\cdot I_{1}=\frac{g\mu_{B}a_{1}}{2}(Q_{1}^{2}-S_{1}^{2}-I_{1}^{2})\;, \end{array}$$ (18)

where $S_{1}^{2}$ and $I_{1}^{2}$ are the total angular momentum operators of the electron and nucleus on the first radical, respectively, both having an eigenvalue of $\frac{3}{4}\hbar^{2}$. $Q_{1}^{2}={\left(S_{1}+I_{1}\right)}^{2}$ is the total spin angular momentum of the first radical consisting of an unpaired electron and a magnetic nucleus; the allowed eigenvalues of $Q_{1}^{2}$ are 0ℏ^2^ and 2ℏ^2^. Thus the possible energy states of the first radical are split by the hyperfine interaction in Eq (18), such that one state has the energy $E_{1}=-\frac{3}{4}g\mu_{B}a_{1}$, corresponding to the eigenvalue 0ℏ^2^ of **Q**^2^, and three states have the energy $E_{2}=\frac{1}{4}g\mu_{B}a_{1}$, corresponding to eigenvalue 2ℏ^2^ of **Q**^2^. The transition frequency between these energy states is therefore:

$$\begin{array}{c} \nu =\frac{E_{2}-E_{1}}{2\pi \hbar }+\Delta \nu =\frac{g\mu_{B}}{2\pi \hbar }a_{1}+\Delta \nu \approx 28\frac{\text{MHz}}{\text{mT}}a_{1}+\Delta \nu , \end{array}$$ (19)

where Δ*ν* is a contribution from the static external magnetic field, which is needed because only the hyperfine interactions were included in *E*_1_ and *E*_2_ while the Zeeman interaction also impacts on the energy difference between the possible states in the radical; Δ*ν* is approximately 0.7 MHz at *B*_0_ = 50 *μ*T. Note that the form of Eq (19) only holds when the static magnetic field is weak compared to the hyperfine interactions, such that it only contributes the small perturbation Δ*ν*; a more rigorous treatment of the impact of the static external magnetic field on the transition frequencies is much more involved. The correspondence between transition frequency and the isotropic hyperfine coupling manifests itself in Fig 5: for *a*_1_ = 0.25 mT one observes a large change in the singlet yield at 0.25 ⋅ 28 MHz + 0.7 MHz = 7.7 MHz, for *a*_1_ = 0.75 mT at 21.7 MHz, and for *a*_1_ = 1.50 mT at 42.7 MHz, as indicated by the dashed lines in Fig 5. For all values of *a*_1_, the probability of forming the singlet product additionally have a large change at 28.7 MHz. This additional feature is caused by the second radical, which has a magnetic nucleus with a fixed isotropic hyperfine coupling of 1 mT.

The simple correspondence between hyperfine interactions and the frequency-dependence of the singlet yield change become more complicated when multiple magnetic nuclei reside on the same radical, due to second-order interactions where nuclei may interact with each other through the hyperfine interactions with the unpaired electron. It should nevertheless be clear from Fig 5 that the hyperfine interactions in a radical pair are crucial in determining whether an RF magnetic field might influence a radical pair reaction, and therefore the most important of the internal molecular parameters to be obtained.

### The effect of exchange and dipole-dipole interaction

**Fig 6:**
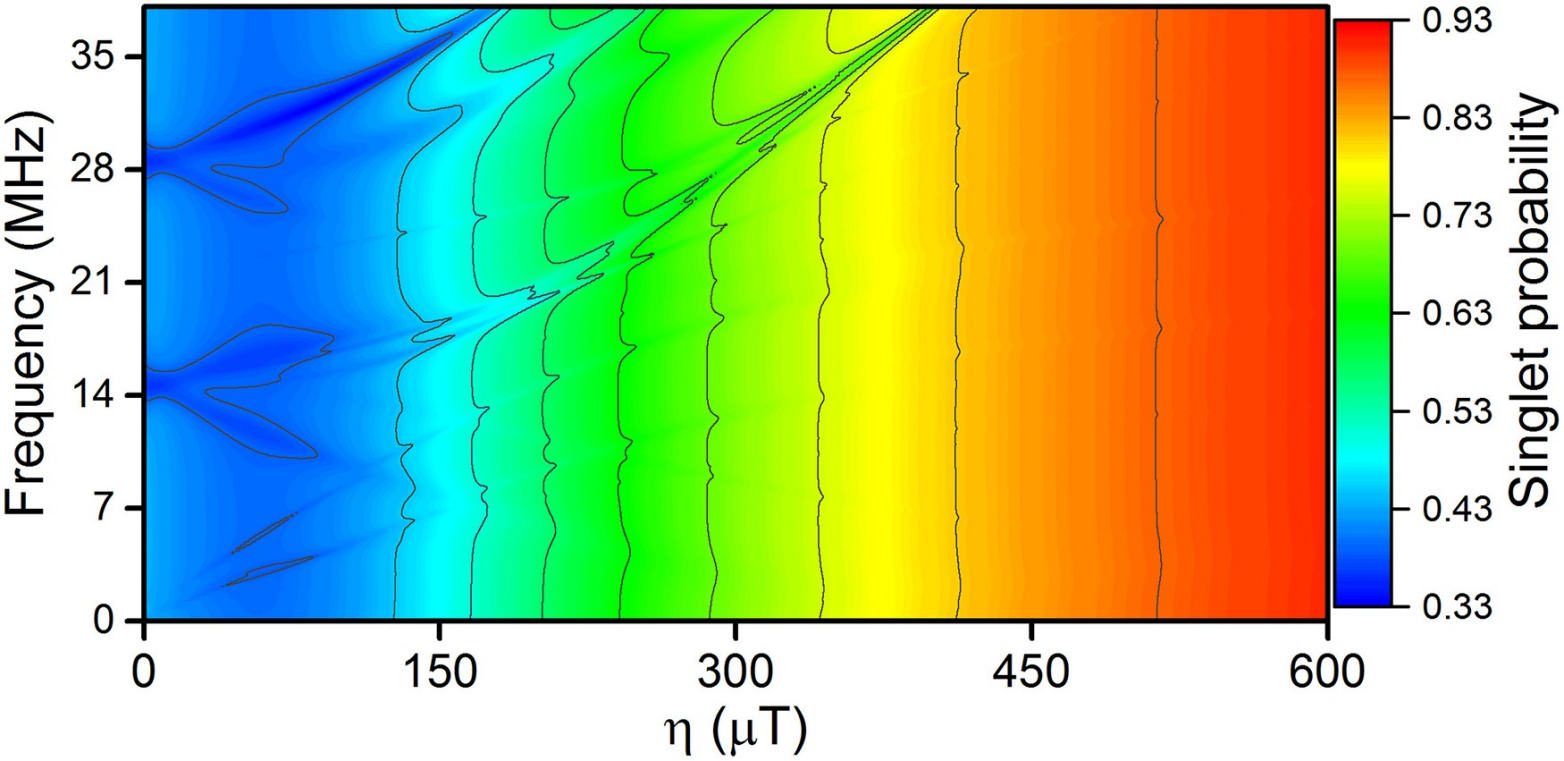
The exchange interaction. The exchange interaction is defiend in Eq (3), but $\eta =-\frac{2J}{g\mu_{B}}$ is used instead of *J* for better comparison with the hyperfine interaction strength. For values of *η* comparable with the hyperfine coupling *a*_1_ = 500 *μ*T the singlet-triplet mixing of the radical pair is supressed, leaving the radical pair largely in its initial singlet state.

Including the exchange interaction, Eq (3), between the radicals of a radical pair mainly results in a less significant response to the RF magnetic field. A strong exchange interaction, i.e. a large value of *J*, supresses the singlet-triplet mixing in the radical pair, which in the simple model radical pair leads to a very high singlet yield. This effect of the exchange interaction is illustrated in Fig 6 as a function of

$$\begin{array}{c} \eta =-\frac{2J}{g\mu_{B}}, \end{array}$$ (20)

instead of *J*, to allow easier comparison with the hyperfine interactions.

The magnetic dipole-dipole interaction between the radicals, Eq (4), has a similar effect to the exchange interaction, with the tensor **D** playing the role of the interaction strength in a similar fashion as *J* for the exchange interaction. The main difference between these two inter-radical interactions is the tensorial nature of **D** which allows for an anisotropic interaction, but ultimately they both just suppress singlet-triplet mixing and hence the RF magnetic field effects. Thus, as depicted in the workflow diagram in Fig 1, there will be no RF magnetic field effect if *J* or **D** is too large, as illustrated in Fig 6, i.e. the peculiarities in the singlet probability vanish when *η* reaches the same order of magnitude as the hyperfine interactions.

### Radical pair lifetime

Whether an RF magnetic field has an impact on the radical pair highly depends on how much time the RF field affects the system, as well as the intensity of the RF field. This is also emphasized in the workflow in Fig 1, which indicate that no RF magnetic field effect is expected once *τ*_*RF*_ ≫ *τ*_0_. Here *τ*_*RF*_ is defined in Eq (2) while the radical pair lifetime *τ*_0_ depends on the reaction rate constants, *k*_*S*_ and *k*_*T*_. In all the model calculations carried out so far, it is assumed that *k*_*S*_ = *k*_*T*_ = *k*, i.e. that there is only a spin-independent reaction with rate constant *k*, leading to a radical pair lifetime of *τ*_0_ = 1/*k*.

**Fig 7:**
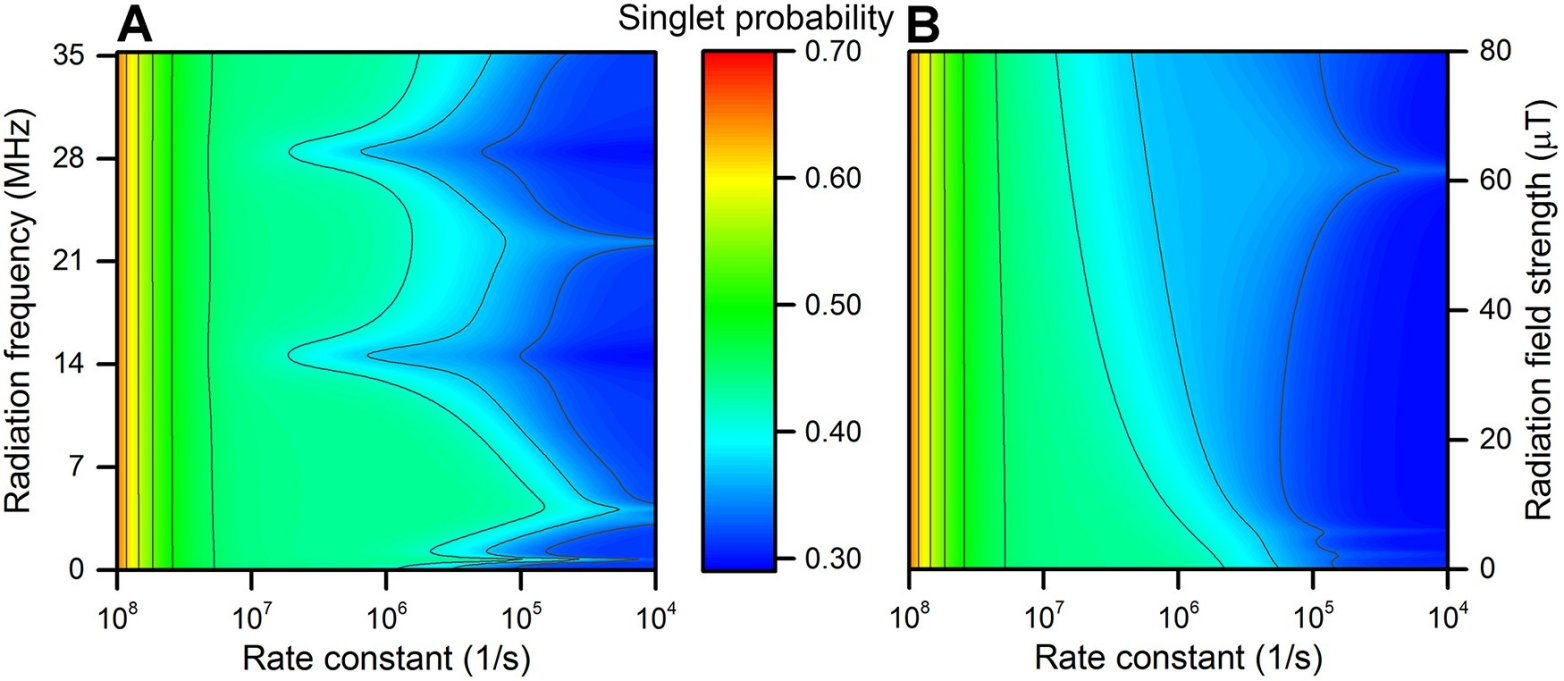
Singlet product probability dependence on *k* (*τ*_0_) and *B*_1_ (*τ*_*RF*_). The singlet product probability is calculated assuming spin-independent kinetics, i.e. *k* = *k*_*S*_ = *k*_*T*_. **A**: The dependence of the reaction probability on RF frequency and reaction rate constant, with *B*_1_ = 50 *μ*T and therefore *τ*_*RF*_ = 714 ns. **B**: The dependence of the reaction probability on RF field intensity and reaction rate constant, with *ω* = 14.4 MHz corresponding to the significant singlet yield change seen in **A** and Fig 4A. The calculations assume *B*_0_ = 50 *μ*T and *a*_1_ = 0.5 mT.

Fig 7 illustrates that the most prominent RF magnetic field effects on the singlet yield requires a lifetime of the radical pair in the range of 100 ns—100 *μ*s. These lifetimes are characteristic for an RF field of 50 *μ*T intensity, and *τ*_*RF*_ would be somewhat different for e.g. a 5 *μ*T RF field since such a weaker RF magnetic field would require more time to influence the radical pair dynamics.

The singlet yield tends towards a value of 0.25 as *τ*_0_ increases, whereas high rate constants result in very high singlet yields. For example, a singlet product probability of about 0.65 was obtained for *k* = 100 *μ*s^−1^, since the radical pair is initially assumed to be generated in the singlet state and the probability for it to react chemically before being converted to the triplet state is high. In general this happens when the time scale for singlet-triplet mixing in a radical pair becomes much longer than its lifetime, *τ*_mix_ ≫ *τ*_0_, i.e. singlet-triplet mixing takes too long for it to happen before most of the system has already chemically reacted, as is seen in Fig 7 for rate constants *k* > 10^7^ s^−1^.

### Modelling a more complex radical pair

As stated earlier, the hyperfine couplings in a radical pair has typically a more profound influence on the RF magnetic field response than any of the other internal molecular parameters, provided *J* and **D** are not too large or *τ*_*RF*_ ≫ *τ*_0_ to prevent any RF magnetic field effects in the first place. Thus it is instructive to see how a radical pair with more hyperfine interactions can be studied. Since it has been previously suggested that RF effects in a $\left[{\text{FAD}}^{•-}\ldots {\text{O}}_{2}^{•-}\right]$ radical pair might lead to increased intracellular superoxide levels [1, 2, 4], this particular system will be considered. It should thus be noted that while the superoxide radical has no hyperfine interactions, the FAD^•−^ radical contains (i) many nuclei on the same radical; (ii) nitrogen nuclei with a spin of 1; (iii) sets of magnetically equivalent nuclei, e.g. the three H8 methyl protons.

The full FAD^•−^ radical contains 15 magnetic nuclei (4 nitrogens and 11 protons), and such a high number of magnetic nuclei can be difficult to handle computationally. Luckily it normally suffices to consider only a subset of the magnetic nuclei, since the magnetic nuclei with the smallest hyperfine couplings only would contribute smaller perturbations to the action spectrum. Thus the 8 magnetic nuclei with the largest hyperfine couplings would make up a representative model of the FAD^•−^ radical.

**Fig 8:**
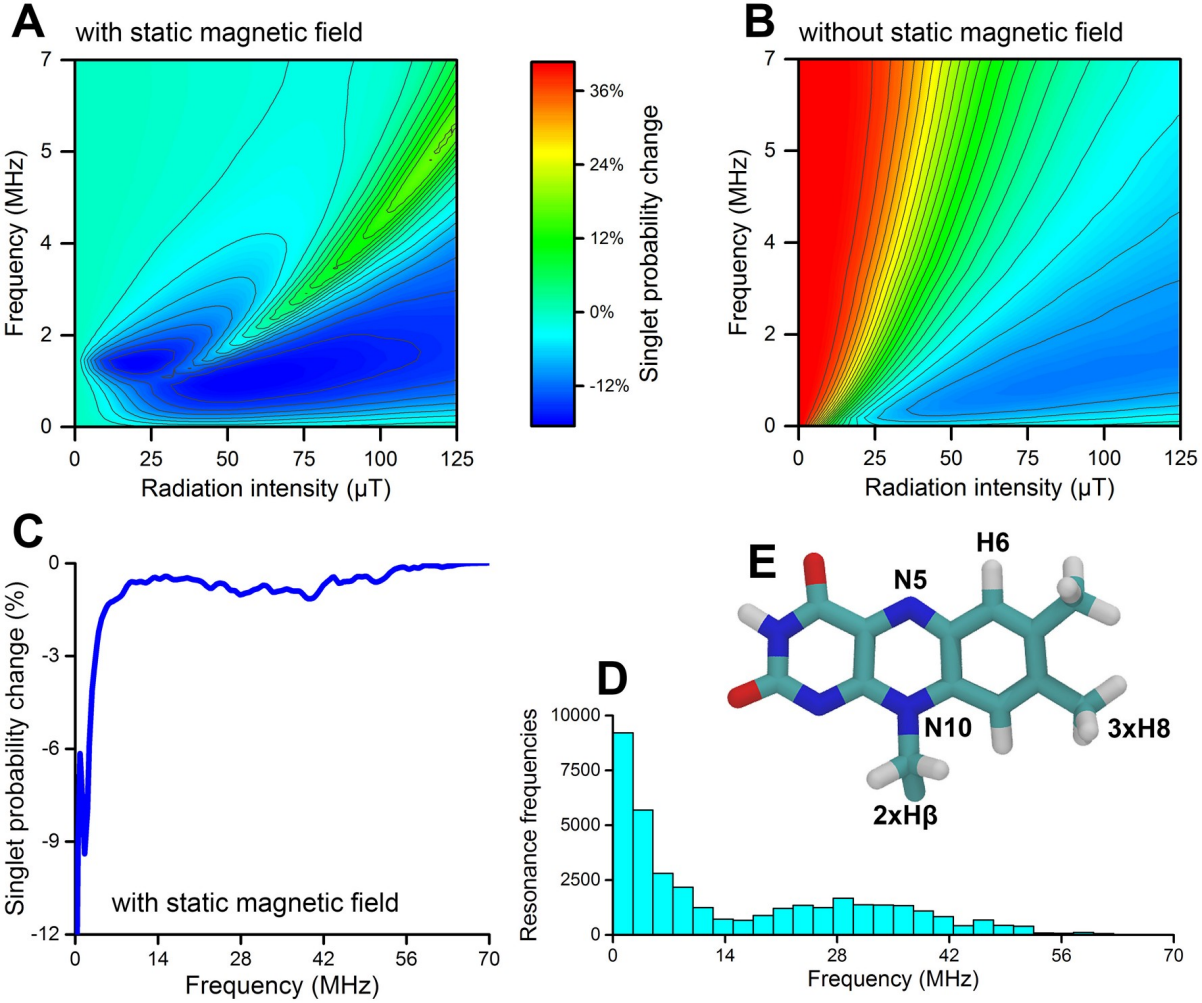
Characterization of RF effects in the $\left[{\text{FAD}}^{•-}\ldots {\text{O}}_{2}^{•-}\right]$ radical pair. **A-C**: The singlet product probability change was calculated for a model including 8 isotropic hyperfine interactions on FAD^•−^ and none on ${\text{O}}_{2}^{•-}$, with (**A** and **C**) or without (**B**) a static external magnetic field of *B*_0_ = 50 *μ*T along the *z*-axis. All calculations assumed *k*_*S*_ = *k*_*T*_ = 1 *μ*s^−1^. Once the static external magnetic field is present, significant singlet probability changes are only seen for RF frequencies below 7 MHz as illustrated in **C**. **D**: The resonance frequencies of the system calculated from the eigenvalues of the spin Hamiltonian. **E**: The nuclei N5, N10, H6, 3 × H8 and 2 × H*β* on FAD were used.

The radical pair is assumed to be produced in the singlet state, and assume that a reaction to form H_2_O_2_ may happen only from the singlet state, while superoxide radicals in a triplet state might escape without forming H_2_O_2_; thus singlet (H_2_O_2_) and triplet (superoxide) products are defined for this radical pair example. In an experiment, the measured amounts of H_2_O_2_ and superoxide provides the quantities $\bar{\langle \Phi_{S}\rangle }$ and $\bar{\langle \Phi_{T}\rangle }$, respectively, and Eq (16) provides the fractions for these two products, 〈Φ_*S*_〉 and 〈Φ_*T*_〉. If the experiment is performed twice, with and without a RF magnetic field, the average singlet yield change, Δ〈Φ_*S*_〉, can then be obtained; it is simply the change in the amount of H_2_O_2_ produced in the experiment with RF magnetic fields, compared to the experiment without RF magnetic fields, and this is exactly the quantity that was computed in Fig 8A–8C.

The presence of even a weak static magnetic field may have a significant effect on the quantum yields, as illustrated by Fig 8A and 8B; the static magnetic field changes the energy levels of the spin states in the radical pair, thereby changing the resonance frequencies of the spin system. For a complex radical like FAD^•−^, which has many hyperfine interactions, this Zeeman-splitting of the energy levels by a static magnetic field is unlikely to be significant. For superoxide, on the other hand, one of the simplest possible radicals, void of any hyperfine interactions, a notable effect would be expected: in the absence of any coupling between the two unpaired electrons (that is, no exchange or magnetic dipole-dipole interaction), the unpaired electron in the superoxide radical can be considered a “free electron”. Such free electrons are known to undergo Larmor precession in a static magnetic field [44], with the precession frequency given by 1.4 MHz in a static magnetic field of 50 *μ*T. Since the main difference between Fig 8A and 8B are seen near 1.4 MHz, this Zeeman-resonance in superoxide seems to be the main cause of the static field effect.

With the more complex model system defined, it would be straightforward to calculate the action of the RF magnetic field as in Fig 4, and the results of such calculations are shown in Fig 8A–8C. Such a comprehensive analysis may not be necessary, particularly if one is interested in RF magnetic fields with a specific frequency *ν*_*RF*_; a simple analysis of the radical pair system can determine its acceptable resonance frequencies, and unless *ν*_*RF*_ is close to any of these frequencies there can be no effect of the RF magnetic field, as illustrated in Fig 1. The resonance frequencies are obtained from the eigenvalues of the spin Hamiltonian for the radical pair system, as the difference between any pair of its eigenvalues. For a Hamiltonian with *n* eigenvalues there will thus be *n*(*n* − 1) resonance frequencies, where *n* may potentially be a rather large number, being determined by the type and number of magnetic nuclei in the radicals. A histogram of the number of resonance frequencies would, therefore, be a simple way to represent them, as is illustrated in Fig 8D for the FAD^•−^ radical. Note that there is a certain similarity between Fig 8C and 8D as each peculiarity in Fig 8C is caused by excitation of specific resonance frequencies. In particular, there is a significant change in the singlet product probability at low frequencies, which is expected since any resonance frequency is calculated as the difference between two energy levels: the largest resonance frequency just above 50 MHz is only about a factor of 2 larger than the largest resonance frequency in the two-nucleus systems studied previously, while the number of energy levels (spin states) has increased from 16 (two electronic spins and two spin-1/2 nuclei) to 6528 (two electronic spins, 6 protons and 2 nitrogen nuclei). The energy levels must therefore be packed much closer to each other, which means that the energy difference between a pair of energy levels will in many cases be very small, leading to a small resonance frequency. As seen in Fig 5, singlet yield change as a function of the oscillation frequency of the RF magnetic field follows a Lorentzian distribution centered around a specific resonance frequency. In other words, not only does the resonance frequencies affect radical pairs, but oscillation frequencies near a given resonance frequency might also have an effect, although the effect is smaller for frequencies further away from a resonance frequency, as dictated by the Lorentzian distribution. The full-width at half-maximum (FWHM) of the Lorentzian distribution depends on the lifetime of the radical pair, and the effect is, therefore, known as lifetime broadening [61]. Due to lifetime broadening, RF magnetic fields of any frequency below a few MHz, dependending on the radical pair lifetime, will, therefore, significantly affect the singlet product probability. This may be considered a general feature of radical pairs with many hyperfine interactions, with the radical pair lifetime being the crucial parameter due to the lifetime broadening.

## Discussion

Determining whether radical pairs residing in a biological environment are susceptible to RF magnetic field effects is no simple matter, but the presented workflow outlines the steps necessary to produce a realistic computational model of ensembles of such radical pairs, as well as the interpretation of calculation results in terms of physical observables. Such a computational approach has the predictive power necessary to evaluate the possible health effects of RF magnetic fields.

The Liouville-von Neumann equation allows to describe the dynamics of ensembles of transient intracellular radical pairs, and permits predicting their response to external RF magnetic fields. Note that only the magnetic aspect of the electromagnetic radiation has been considered here; electric fields do not directly interact with spins, except for the nuclear quadrupole interaction for nuclei with a spin of at least 1, and any indirect effects by e.g. a displacement in the electronic density would be negligible compared to the magnetic interactions. The calculation examples in the present study employed the rotating reference frame approach to establish the characteristic change of the radical pair singlet product probability, which would characterize such a response. This theoretical description was chosen due to its simplicity, which served well to demonstrate many features of RF magnetic field responses in model radical pair ensembles.

The change in the probability of the singlet product caused by an RF magnetic field has been shown in model calculations and previous studies [13, 28, 32, 51] to depend on the structure of the molecular system hosting the radical pair, described mainly through the hyperfine interactions. The generic two-nuclei model displayed a very simple relationship between the peculiarities in the singlet yield change and the RF magnetic field frequency, and similar relationships may also be found for more realistic models containing more magnetic nuclei, although they would be much more complex due to the larger number of nuclear spin states as illustrated for the $\left[{\text{FAD}}^{•-}\ldots {\text{O}}_{2}^{•-}\right]$ radical pair. The computations predict that for significant RF field-induced effects to happen within the 1-50 MHz range with RF field strengths below 100 *μ*T, the recombination and chemical reactions of the radical pair should occur with rate constants in the interval of 10^4^-10^7^ s^−1^. Although the present investigation focuses on a simplified model system, these limits on the rate constants are expected not to be much different for a more realistic radical pair system since they depend on the time scale for singlet-triplet mixing, which in turn depends on the strength of the hyperfine interactions; even though the hyperfine interactions in a real molecular system could be larger than those of the studied model system, they are expected to be of the same order of magnitude. It should be noted, however, that the rate constants for chemical reactions involving singlet and triplet states are in general not equal, since recombination may happen either only from the singlet state or only from the triplet state [54]. Although different reaction rate constants for singlet and triplet were not explored in the present study, it is already supported by the formalism described above, see e.g. Eq (9).

The workflow described here represents a general framework that can be applied to many different physical systems, not even limited to radical pairs. In fact many complexes involving metal ions would posses a number of unpaired electrons, which could take the role of one of the radicals in a radical pair, the main difference being the spin quantum number. The main challenge that remains in order to explain the observed RF magnetic field effects in cells is, therefore, to find cellular processes that could be affected, and design experiments to obtain the magnitude of any RF field effects. Theoretical calculations as those presented here can both aid in explaining observed effects of RF fields, and make predictions about frequency ranges that deserve experimental investigation in order to observe an effect.

## Supporting information

S1 Fig The three Euler angles *α*, *β* and *γ*. First a rotation about the *z*-axis by angle *α* is performed, then a rotation about the initial *x*-axis by the angle *β*, and finally a rotation about the *z*-axis by the angle *γ*.
(PDF)

S1 File Spin systems and mixing of states. The summary provides a short introduction to the essential concepts of spin dynamics used in the paper.
(PDF)

S2 File Rotation matrices. The basic concepts of rotation matrices that describe transformation from the molecular frame of reference to the laboratory frame.
(PDF)

## References

[1] Usselman RJ, Chavarriaga C, Castello PR, Procopio M, Ritz T, Dratz EA, et al The Quantum Biology of Reactive Oxygen Species Partitioning Impacts Cellular Bioenergetics. Sci Rep. 2016;6:38543 10.1038/srep38543 27995996 PMC5172244

[2] Usselman RJ, Hill I, Singel DJ, Martino CF. Spin biochemistry modulates reactive oxygen species (ROS) production by radio frequency magnetic fields. PLoS One. 2014;9:e93065 10.1371/journal.pone.0093065 24681944 PMC3969378

[3] Castello PR, Hill I, Sivo F, Portelli L, Barnes F, Usselman R, Martino CF. Inhibition of cellular proliferation and enhancement of hydrogen peroxide production in fibrosarcoma cell line by weak radio frequency magnetic fields Bioelectromagnetics. 2014;35:598–602. 10.1002/bem.21858 25251337

[4] Naarala J, Kesari KK, McClure I, Chavarriaga C, Juutilainen J, Martino CF. Direction-Dependent Effects of Combined Static and ELF Magnetic Fields on Cell Proliferation and Superoxide Radical Production. Biomed Res Int. 2017;2017:5675086 10.1155/2017/5675086 28497056 PMC5405400

[5] Steiner UE, Ulrich T. Magnetic field effects in chemical kinetics and related phenomena. Chem Rev. 1989;89:51–147. 10.1021/cr00091a003

[6] Hore PJ, Mouritsen H. The Radical-Pair Mechanism of Magnetoreception. Annu Rev Biophys. 2016;45:299–344. 10.1146/annurev-biophys-032116-094545 27216936

[7] Timmel CR, Till U, Brocklehurst B, Mclauchlan KA, Hore PJ. Effects of weak magnetic fields on free radical recombination reactions. Mol Phys. 1998;95:71–89. 10.1080/09553000050176270 11098854

[8] Timmel CR, Cintolesi F, Brocklehurst B, Hore PJ. Model calculations of magnetic field effects on the recombination reactions of radicals with anisotropic hyperfine interactions. Chem Phys Lett. 2001;334:387–395. 10.1016/S0009-2614(00)01436-6

[9] Pedersen JB, Nielsen C, Solov’yov IA. Multiscale description of avian migration: from chemical compass to behaviour modeling. Sci Rep. 2016;6:36709 10.1038/srep36709 27830725 PMC5103213

[10] Hui SY. Planar Wireless Charging Technology for Portable Electronic Products and Qi. Proceedings of the IEEE. 2013;101:1290–1301. 10.1109/JPROC.2013.2246531

[11] Covic GA, Boys JT. Inductive Power Transfer. Proceedings of the IEEE. 2013;101:1276–1289. 10.1109/JPROC.2013.2244536

[12] Rodgers CT, Henbest KB, Kukura P, Timmel CR, Hore PJ. Low-field optically detected EPR spectroscopy of transient photoinduced radical pairs. J Phys Chem A. 2005;109:5035–5041. 10.1021/jp050765z 16833855

[13] Rodgers CT, Wedge CJ, Norman SA, Kukura P, Nelson K, Baker N, et al Radiofrequency polarization effects in zero-field electron paramagnetic resonance. Phys Chem Chem Phys. 2009;11:6569–6572. 10.1039/b906102a 19639131

[14] Henbest KB, Kukura P, Rodgers CT, Hore PJ, Timmel CR. Radio Frequency Magnetic Field Effects on a Radical Recombination Reaction: A Diagnostic Test for the Radical Pair Mechanism. J Am Chem Soc. 2004;126:8102–8103. 10.1021/ja048220q 15225036

[15] Solov’yov IA, Chandler DE, Schulten K. Magnetic field effects in Arabidopsis thaliana Cryptochrome-1. Biophys J. 2007;92:2711–2726. 10.1529/biophysj.106.097139 17259272 PMC1831705

[16] Solov’yov IA, Schulten K. Reaction kinetics and mechanism of magnetic field effects in cryptochrome. J Phys Chem B. 2012;116:1089–1099. 10.1021/jp209508y 22171949 PMC3266978

[17] Kattnig DR, Sowa JK, Solov’yov IA, Hore PJ. Electron spin relaxation can enhance the performance of a cryptochrome-based magnetic compass sensor. New J Phys. 2016;18:063007. 10.1039/c5cp06731f 27020113

[18] Kattnig DR, Solov’yov IA, Hore PJ. Electron spin relaxation in cryptochrome-based magnetoreception. Phys Chem Chem Phys. 2016;70:12443–12456. 10.1039/C5CP06731F 27020113

[19] Brocklehurst B. Magnetic fields and radical reactions: recent developments and their role in Nature. Chem Soc Rev. 2002;31:301–311. 10.1039/b107250c 12357727

[20] Haberkorn R. Density matrix description of spin-selective radical pair reactions. Mol Phys. 1976;32:1491–1493. 10.1080/00268977600102851

[21] Solov’yov IA, Schulten K. Magnetoreception through Cryptochrome may involve superoxide. Biophys J. 2009;96:4804–4813. 10.1016/j.bpj.2009.03.048 19527640 PMC2712043

[22] Ritz T, Wiltschko R, Hore PJ, Rodgers CT, Stapput K, Thalau P, et al Magnetic compass of birds is based on a molecule with optimal directional sensitivity. Biophys J. 2009;96:3451–3457. 10.1016/j.bpj.2008.11.072 19383488 PMC2718301

[23] Barragan AM, Schulten K, Solov’yov IA. Mechanism of the Primary Charge Transfer Reaction in the Cytochrome bc1 Complex. J Phys Chem B. 2016;120:11369–11380. 10.1021/acs.jpcb.6b07394 27661199 PMC5721205

[24] Salo AB, Husen P, Solov’yov IA. Charge transfer at the Qo-site of the cytochrome bc1 complex leads to superoxide production. J Phys Chem B. 2017;121:1771–1782. 10.1021/acs.jpcb.6b10403 27983847

[25] Husen P, Solov’yov IA. Spontaneous Binding of Molecular Oxygen at the Qo-Site of the bc1 Complex Could Stimulate Superoxide Formation. J Am Chem Soc. 2016;138:12150–12158. 10.1021/jacs.6b04849 27447781

[26] Husen P, Solov’yov IA. Mutations at the Qo site of the cytochrome bc1 complex strongly affect oxygen binding. J Phys Chem B. 2017;121:3308–3317. 10.1021/acs.jpcb.6b08226 27748117

[27] Barragan AM, Crofts AR, Schulten K, Solov’yov IA. Identification of Ubiquinol Binding Motifs at the Qo-Site of the Cytochrome bc1 Complex. J Phys Chem B. 2015;119:433–447. 10.1021/jp510022w 25372183 PMC4297238

[28] Timmel CR, Hore PJ. Oscillating magnetic field effects on the yields of radical pair reactions. Chem Phys Lett. 1996;257:401–408. 10.1016/0009-2614(96)00466-6

[29] Canfield JM, Belford RL, Debrunner PG, Schulten KJ. A perturbation theory treatment of oscillating magnetic fields in the radical pair mechanism. Chem Phys. 1994;182:1–18. 10.1016/0301-0104(93)E0442-X

[30] Canfield JM, Belford RL, Debrunner PG, Schulten KJ. A perturbation treatment of oscillating magnetic fields in the radical pair mechanism using the Liouville equation. Chem Phys. 1995;195:59–69. 10.1016/0301-0104(95)00049-T

[31] Hiscock HG, Kattnig DR, Manolopoulos DE, Hore PJ. Floquet theory of radical pairs in radiofrequency magnetic fields. J Chem Phys. 2016;145:124117 10.1063/1.4963793 27782620

[32] Wang K, Ritz T. Zeeman resonances for radical-pair reactions in weak static magnetic fields. Mol Phys. 2006;104:1649–1658. 10.1080/00268970600564869

[33] Hohwy M, Bildsøe H, Jakobsen HJ, Nielsen NC. Efficient Spectral Simulations in NMR of Rotating Solids. The *γ*-COMPUTE Algorithm. J Magn Reson. 1999;136:6–14. 10.1006/jmre.1998.1593 9887283

[34] Nielsen C, Kattnig DR, Sjulstok E, Hore PJ, Solov’yov IA. Ascorbic acid may not be involved in cryptochrome-based magnetoreception. J R Soc Interface. 2017;14:20170657 10.1098/rsif.2017.0657 29263128 PMC5746572

[35] Bittner ER. Quantum Dynamics: Applications in Biological and Materials Systems. CRC Press, Taylor & Francis Group; 2010.

[36] Solov’yov IA, Chang PY, Schulten K. Vibrationally Assisted Electron Transfer Mechanism of Olfaction: Myth or Reality? Phys Chem Chem Phys. 2012;14:13861–13871. 10.1039/c2cp41436h 22899100 PMC3478898

[37] Marcus RA. Electron-transfer reactions in chemistry—theory and experiment. Rev Mod Phys. 1993;65:599–610. 10.1103/RevModPhys.65.599

[38] Miyashita O, Go N. Reorganization Energy of Protein Electron Transfer Reaction: Study with Structural and Frequency Signature. J Phys Chem B. 2000;104(31):7516–7521. 10.1021/jp000865z

[39] Reese A, List NH, Kongsted J, Solov’yov IA. How Far Does a Receptor Influence Vibrational Properties of an Odorant? PLoS ONE. 2016;11 10.1371/journal.pone.0152345 PMC4807836 27014869

[40] Lüdemann G, Solov’yov IA, Kubař T, Elstner M. Solvent driving force ensures fast formation of a persistent and well-separated radical pair in plant cryptochrome. J Am Chem Soc. 2015;137:1147–1156. 10.1021/ja510550g 25535848

[41] Grissom CB. Magnetic Field Effects in Biology: A Survey of Possible Mechanisms with Emphasis on Radical-Pair Recombination. Chem Rev. 1995;95:3–24. 10.1021/cr00033a001

[42] Frisch MJ, Trucks GW, Schlegel HB, Scuseria GE, Robb MA, Cheeseman JR, et al. Gaussian 09 Revision D.01; 2009.

[43] Aidas K, Angeli C, Bak KL, Bakken V, Bast R, Boman L, et al The Dalton quantum chemistry program system. Wiley Interdisciplinary Reviews: Computational Molecular Science. 2014;4:269–284. 10.1002/wcms.1172 25309629 PMC4171759

[44] Griffith DJ. Introduction to Quantum Mechanics, 2nd ed Pearson Prentice Hall; 2005.

[45] Atherton NM. Principles of Electron Spin Resonance. Ellis Horwood PTR Prentice Hall; 1993.

[46] Levitt MH. Spin Dynamics—Basics of Nuclear Magnetic Resonance. Wiley and Sons; 2001.

[47] Breuer HP, Petruccione F. The Theory of Open Quantum Systems. Oxford University Press; 2007.

[48] Worster S, Kattnig DR, Hore PJ. Spin relaxation of radicals in cryptochrome and its role in avian magnetoreception. J Chem Phys. 2016;145:035104 10.1063/1.4958624 27448908

[49] Nielsen C, Solov’yov IA. MolSpin—Flexible and Extensible General Spin Dynamics Software. 2019. 10.1063/1.5125043 31757147

[50] https://viking.sdu.dk/.

[51] Hiscock HG, Mouritsen H, Manolopoulos DE, Hore PJ. Disruption of Magnetic Compass Orientation in Migratory Birds by Radiofrequency Electromagnetic Fields. Biophys J. 2017;113:1475–1484. 10.1016/j.bpj.2017.07.031 28978441 PMC5627152

[52] Sakurai JJ, Napolitano JJ. Modern Quantum Mechanics, 2nd ed Pearson; 2014.

[53] Landau LD, Lifshitz EM. Quantum Mechanics: Non-Relativistic Theory. Elsevier Butterworth-Heinemann; 1981.

[54] Lewis AM, Manolopoulos DE, Hore PJ. Asymmetric recombination and electron spin relaxation in the semiclassical theory of radical pair reactions. J Chem Phys. 2014;141:044111 10.1063/1.4890659 25084885

[55] Merzbacher E. Quantum Mechanics, 3rd ed Wiley and Sons; 1998.

[56] Nielsen MA. Quantum Computation and Quantum Information. Cambridge University Press; 2010.

[57] Ritz T, Adem S, Schulten K. A Model for Photoreceptor-Based Magnetoreception in Birds. Biophys J. 2000;78:707–718. 10.1016/S0006-3495(00)76629-X 10653784 PMC1300674

[58] Hansen MJ, Pedersen JB. Recombination yield of geminate radical pairs in low magnetic fields. Chem Phys Lett. 2002;361:219–225. 10.1016/S0009-2614(02)00724-8

[59] Maeda K, Neil SRT, Henbest KB, Weber S, Schleicher E, Hore PJ, et al Following Radical Pair Reactions in Solution: A Step Change in Sensitivity Using Cavity Ring-Down Detection. J Am Chem Soc. 2011;133:17807–17815. 10.1021/ja206783t 21932826

[60] Maeda K, Storey JG, Liddell PA, Gust D, Hore PJ, Wedge CJ, et al Probing a chemical compass: novel variants of low-frequency reaction yield detected magnetic resonance. Phys Chem Chem Phys. 2015;17:3550–3559. 10.1039/c4cp04095c 25537133

[61] Demtröder W. Laser Spectroscopy 1: Basic Principles, 5th ed Springer; 2014.

