# Supplementary material for: Towards predicting intracellular radiofrequency radiation effects
Source: PLoS One. 2019 Mar 14;14(3):e0213286. doi: 10.1371/journal.pone.0213286 (PMC6417702; doi:10.1371/journal.pone.0213286)
Supplement: S1 Fig — First a rotation about the z-axis by angle α is performed, then a rotation about the initial x-axis by the angle β, and finally a rotation about the z-axis by the angle γ. (PDF) [file pone.0213286.s001.pdf]

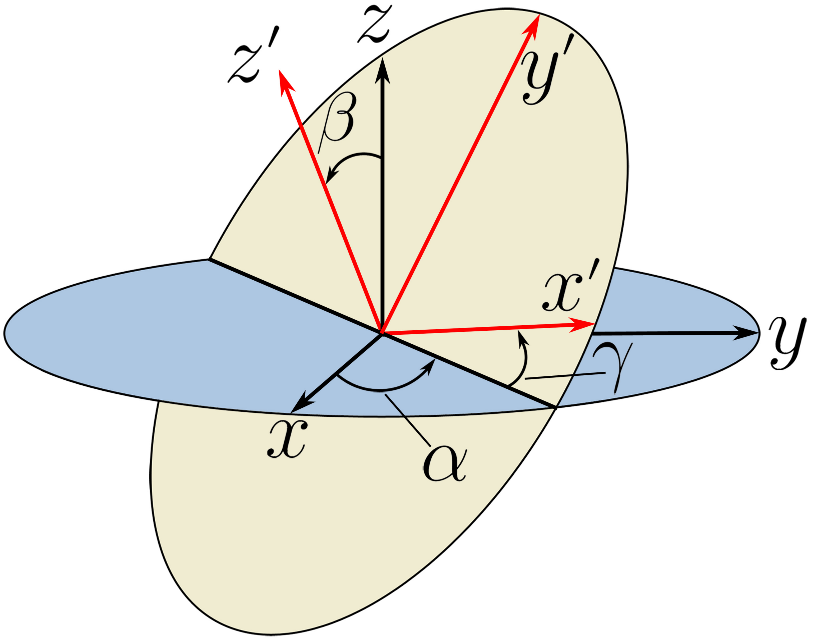

S1 Fig. The three Euler angles α, β and γ. First a rotation about the z-axis by angle α is performed, then a rotation about the initial x-axis by the angle β, and finally a rotation about the z-axis by the angle γ.
