# Supplementary material for: Towards predicting intracellular radiofrequency radiation effects
Source: PLoS One. 2019 Mar 14;14(3):e0213286. doi: 10.1371/journal.pone.0213286 (PMC6417702; doi:10.1371/journal.pone.0213286)
Supplement: S1 File — The summary provides a short introduction to the essential concepts of spin dynamics used in the paper. (PDF) [file pone.0213286.s002.pdf]

S1 Spin systems and mixing of states

The summary below provides a short introduction to the essential concepts of spin dynamics used in the main paper. More information can be found in various basic quantum mechanics textbooks, see e.g. (1–4).

S1.1 Spin states and operators

The quantum state of a spin-1/2 particle, such as an electron, can be described in terms of two spin states. Normally these two states are chosen to be the spin up state, denoted $|\alpha\rangle$, and spin down state, $|\beta\rangle$. A linear combination (superposition) of these two spin states is also a valid spin state and can be written as:

$$|\psi\rangle = a\,|\alpha\rangle + b\,|\beta\rangle, \tag{S1}$$

with the only constraint for $|\psi\rangle$ to be normalized, i.e.:

$$a^2 + b^2 = 1\,. \tag{S2}$$

Here a and b in general are some complex numbers. If the spin state of $|\psi\rangle$ was measured experimentally (with respect to the spin up/spin down basis used here), there would be a probability of $|a|^2$ to find the particle in the state $|\alpha\rangle$, and a probability of $|b|^2$ to find the particle in the $|\beta\rangle$ spin state. Note that the normalization condition ensures that the total probability of these two possible outcomes of a measurement is 1.

Now consider a second spin-1/2 particle, e.g. assume a radical pair. In that case one now has four basis states since any combination of the two spin states for the first particle

together with any spin state of the second particle is possible, i.e.:

$$|\alpha\alpha\rangle,\ |\alpha\beta\rangle,\ |\beta\alpha\rangle,\ |\beta\beta\rangle\,. \tag{S3}$$

Any normalized linear combination of these spin states is then also a valid spin state, just as for the single-particle case, and there is a special set of two-particle states that are important:

$$|S\rangle = \frac{1}{\sqrt{2}}\left(\,|\alpha\beta\rangle - |\beta\alpha\rangle\right), \qquad |T_+\rangle = |\alpha\alpha\rangle, \tag{S4}$$

$$|T_0\rangle = \frac{1}{\sqrt{2}}\left(\,|\alpha\beta\rangle + |\beta\alpha\rangle\right), \qquad |T_-\rangle = |\beta\beta\rangle\,. \tag{S5}$$

The state $|S\rangle$ is known as the singlet state, and the remaining three states are called triplet states. Some chemical processes occur at different rates depending on whether the radical pair is in a singlet or triplet state, and only the triplet states have a non-zero magnetic moment, such that only the triplet states can be affected by external magnetic fields.

Note that the $|S\rangle$ and $|T_0\rangle$ states are defined as linear combinations of two states from Eq. (S3); these two states cannot be written in terms of a specific spin state of the first spin and a specific spin state of the second spin, but only exist in the combined two-particle Hilbert space. In the $|S\rangle$ and $|T_0\rangle$ states the two spins, therefore, cannot be regarded as two independent particles, and one often denotes these two spins as entangled, or say that $|S\rangle$ and $|T_0\rangle$ as coherent spin states. Such coherences play a crucial role in the appearance of magnetic field effects in radical pairs.

The spin up and spin down states can be described as vectors in a two-dimensional vector space as:

$$|\alpha\rangle = \begin{pmatrix} 1 \\ 0 \end{pmatrix}, \qquad |\beta\rangle = \begin{pmatrix} 0 \\ 1 \end{pmatrix}. \tag{S6}$$

The identity operator, **1**, and spin operators are given by:

$$\mathbf{1} = \begin{pmatrix} 1 & 0 \\ 0 & 1 \end{pmatrix}, \quad \mathbf{S}_x = \frac{\hbar}{2}\begin{pmatrix} 0 & 1 \\ 1 & 0 \end{pmatrix}, \quad \mathbf{S}_y = \frac{\hbar}{2}\begin{pmatrix} 0 & -i \\ i & 0 \end{pmatrix}, \quad \mathbf{S}_z = \frac{\hbar}{2}\begin{pmatrix} 1 & 0 \\ 0 & -1 \end{pmatrix}, \tag{S7}$$

where the three spin matrices without the factor of $\hbar/2$ are known as the Pauli spin matrices. When two particles are present, a state such as $|\alpha\beta\rangle$ can be written in a two-particle Hilbert space in terms of the Kronecker tensor product, $|\alpha\beta\rangle = |\alpha\rangle \otimes |\beta\rangle$ as:

$$|\alpha\alpha\rangle = |\alpha\rangle \otimes |\alpha\rangle = \begin{pmatrix} 1 \cdot \begin{pmatrix} 1 \\ 0 \end{pmatrix} \\ 0 \cdot \begin{pmatrix} 1 \\ 0 \end{pmatrix} \end{pmatrix} = \begin{pmatrix} 1 \\ 0 \\ 0 \\ 0 \end{pmatrix}, \tag{S8}$$

$$|\alpha\beta\rangle = |\alpha\rangle \otimes |\beta\rangle = \begin{pmatrix} 1 \cdot \begin{pmatrix} 0 \\ 1 \end{pmatrix} \\ 0 \cdot \begin{pmatrix} 0 \\ 1 \end{pmatrix} \end{pmatrix} = \begin{pmatrix} 0 \\ 1 \\ 0 \\ 0 \end{pmatrix}, \tag{S9}$$

$$|\beta\alpha\rangle = |\beta\rangle \otimes |\alpha\rangle = \begin{pmatrix} 0 \cdot \begin{pmatrix} 1 \\ 0 \end{pmatrix} \\ 1 \cdot \begin{pmatrix} 1 \\ 0 \end{pmatrix} \end{pmatrix} = \begin{pmatrix} 0 \\ 0 \\ 1 \\ 0 \end{pmatrix}, \tag{S10}$$

$$|\beta\beta\rangle = |\beta\rangle \otimes |\beta\rangle = \begin{pmatrix} 0 \cdot \begin{pmatrix} 0 \\ 1 \end{pmatrix} \\ 1 \cdot \begin{pmatrix} 0 \\ 1 \end{pmatrix} \end{pmatrix} = \begin{pmatrix} 0 \\ 0 \\ 0 \\ 1 \end{pmatrix}. \tag{S11}$$

Hence the singlet state can be written as:

$$|S\rangle = \frac{1}{\sqrt{2}}\left(|\alpha\beta\rangle - |\beta\alpha\rangle\right) = \frac{1}{\sqrt{2}}\left(\begin{pmatrix}0\\1\\0\\0\end{pmatrix} - \begin{pmatrix}0\\0\\1\\0\end{pmatrix}\right) = \begin{pmatrix}0\\\frac{1}{\sqrt{2}}\\-\frac{1}{\sqrt{2}}\\0\end{pmatrix}. \tag{S12}$$

The state $|T_0\rangle$ can be written similarly, and the remaining triplet states are equal to $|\alpha\alpha\rangle$ or $|\beta\beta\rangle$:

$$|T_0\rangle = \begin{pmatrix}0\\\frac{1}{\sqrt{2}}\\\frac{1}{\sqrt{2}}\\0\end{pmatrix}, \quad |T_+\rangle = \begin{pmatrix}1\\0\\0\\0\end{pmatrix}, \quad |T_-\rangle = \begin{pmatrix}0\\0\\0\\1\end{pmatrix}. \tag{S13}$$

Spin operators for the two-particle system can also be derived using the Kronecker tensor

product, e.g.:

$$\mathbf{S}_{2x} = \mathbf{1} \otimes \mathbf{S}_x = \begin{pmatrix} 1 \cdot \begin{pmatrix} 0 & 1 \\ 1 & 0 \end{pmatrix} & 0 \cdot \begin{pmatrix} 0 & 1 \\ 1 & 0 \end{pmatrix} \\ 0 \cdot \begin{pmatrix} 0 & 1 \\ 1 & 0 \end{pmatrix} & 1 \cdot \begin{pmatrix} 0 & 1 \\ 1 & 0 \end{pmatrix} \end{pmatrix} = \begin{pmatrix} 0 & 1 & 0 & 0 \\ 1 & 0 & 0 & 0 \\ 0 & 0 & 0 & 1 \\ 0 & 0 & 1 & 0 \end{pmatrix}, \tag{S14}$$

$$\mathbf{S}_{2y} = \mathbf{1} \otimes \mathbf{S}_y = \begin{pmatrix} 1 \cdot \begin{pmatrix} 0 & -i \\ i & 0 \end{pmatrix} & 0 \cdot \begin{pmatrix} 0 & -i \\ i & 0 \end{pmatrix} \\ 0 \cdot \begin{pmatrix} 0 & -i \\ i & 0 \end{pmatrix} & 1 \cdot \begin{pmatrix} 0 & -i \\ i & 0 \end{pmatrix} \end{pmatrix} = \begin{pmatrix} 0 & -i & 0 & 0 \\ i & 0 & 0 & 0 \\ 0 & 0 & 0 & -i \\ 0 & 0 & i & 0 \end{pmatrix}, \tag{S15}$$

$$\mathbf{S}_{2z} = \mathbf{1} \otimes \mathbf{S}_z = \begin{pmatrix} 1 \cdot \begin{pmatrix} 1 & 0 \\ 0 & -1 \end{pmatrix} & 0 \cdot \begin{pmatrix} 1 & 0 \\ 0 & -1 \end{pmatrix} \\ 0 \cdot \begin{pmatrix} 1 & 0 \\ 0 & -1 \end{pmatrix} & 1 \cdot \begin{pmatrix} 1 & 0 \\ 0 & -1 \end{pmatrix} \end{pmatrix} = \begin{pmatrix} 1 & 0 & 0 & 0 \\ 0 & -1 & 0 & 0 \\ 0 & 0 & 1 & 0 \\ 0 & 0 & 0 & -1 \end{pmatrix}, \tag{S16}$$

and the action of such an operator on a quantum state is $\mathbf{S}_{2z} |\alpha\beta\rangle = (\mathbf{1} |\alpha\rangle) \otimes (\mathbf{S}_z |\beta\rangle)$. As this example illustrates, the Kronecker tensor product can be used to create an operator that only acts on one of the spins, i.e. $\mathbf{S}_{1x} = \mathbf{S}_x \otimes \mathbf{1}$ acts only on the first spin as the identity operator does not affect the second spin, likewise $\mathbf{S}_{2x} = \mathbf{1} \otimes \mathbf{S}_x$ acts only on the second spin, and $\mathbf{S}_{1x}\mathbf{S}_{2y} = \mathbf{S}_x \otimes \mathbf{S}_y$ acts on both spins at the same time. Note that sometimes a total spin operator is used, e.g. $\mathbf{S}_{z,\text{total}} = \mathbf{S}_{1z} + \mathbf{S}_{2z}$, which acts on both spins.

S1.2 Three spin-1/2 particles: Mixing of states

The spin system can be extended with a magnetic nucleus simply by using an additional Kronecker product; assuming for simplicity that the nucleus has a spin of $\hbar/2$. For example,

if the two unpaired electrons are in the $|\alpha\rangle$ state and the magnetic nucleus in the $|\beta\rangle$ state, the full spin state would then be $|\alpha\alpha\beta\rangle = |\alpha\rangle \otimes |\alpha\rangle \otimes |\beta\rangle$. Note that the dimensions of the Hilbert space is now 8 (there exists 8 different states of this type). Also note that one could have chosen to write the same full spin state using singlet/triplet states for the two unpaired electrons of the radical pair as $|T_+\beta\rangle = |T_+\rangle \otimes |\beta\rangle$.

Assume that the radical pair has a simple Hamiltonian with only an isotropic hyperfine interaction, and no other interactions present:

$$\mathbf{H} = g\mu_B a \mathbf{S}_1 \cdot \mathbf{I} = \frac{g\mu_B a}{2}(\mathbf{Q}_1^2 - \mathbf{S}_1^2 - \mathbf{I}^2)\,. \tag{S17}$$

This Hamiltonian is similar to Eq. (18), with $\mathbf{Q} = \mathbf{S}_1 + \mathbf{I}$ where $\mathbf{S}_1$ and $\mathbf{I}$ are the spin operators of the first unpaired electron and the magnetic nucleus, respectively, $g = 2$ is the isotropic Landé g-factor, μ_B the Bohr magneton, and a the hyperfine constant that determines the strength of the hyperfine interaction. The eigenvalues and eigenstates of Eq. (S17) are then:

$$|v_1\rangle = |\alpha\alpha\alpha\rangle, \quad E_1 = \frac{1}{4}g\mu_B a, \tag{S18}$$

$$|v_2\rangle = |\beta\alpha\alpha\rangle, \quad E_2 = \frac{1}{4}g\mu_B a, \tag{S19}$$

$$|v_3\rangle = |\alpha\beta\beta\rangle, \quad E_3 = \frac{1}{4}g\mu_B a, \tag{S20}$$

$$|v_4\rangle = |\beta\beta\beta\rangle, \quad E_4 = \frac{1}{4}g\mu_B a, \tag{S21}$$

$$|v_5\rangle = \frac{1}{\sqrt{2}}(|\alpha\alpha\beta\rangle + |\alpha\beta\alpha\rangle), \quad E_5 = \frac{1}{4}g\mu_B a, \tag{S22}$$

$$|v_6\rangle = \frac{1}{\sqrt{2}}(|\beta\alpha\beta\rangle + |\beta\beta\alpha\rangle), \quad E_6 = \frac{1}{4}g\mu_B a, \tag{S23}$$

$$|v_7\rangle = \frac{1}{\sqrt{2}}(|\alpha\alpha\beta\rangle - |\alpha\beta\alpha\rangle), \quad E_7 = -\frac{3}{4}g\mu_B a, \tag{S24}$$

$$|v_8\rangle = \frac{1}{\sqrt{2}}(|\beta\alpha\beta\rangle - |\beta\beta\alpha\rangle), \quad E_8 = -\frac{3}{4}g\mu_B a\,. \tag{S25}$$

Assuming that a radical pair is generated at the time instace $t = 0$ in the state $|S\alpha\rangle$, one writes this state in terms of the eigenstates:

$$|\psi(0)\rangle = |S\alpha\rangle = \frac{1}{\sqrt{2}}(|\alpha\beta\alpha\rangle - |\beta\alpha\alpha\rangle) = \frac{1}{2}(|v_5\rangle - |v_7\rangle) - \frac{1}{\sqrt{2}}|v_2\rangle\,. \quad \text{(S26)}$$

The time-evolution of the state is governed by the Schrödinger equation:

$$i\hbar\,|\psi(t)\rangle = \mathbf{H}\,|\psi(t)\rangle, \qquad |\psi(t)\rangle = e^{-\frac{i}{\hbar}\mathbf{H}t}\,|\psi(0)\rangle\,. \quad \text{(S27)}$$

Using the eigenstates of $\mathbf{H}$ the solution becomes:

$$|\psi(t)\rangle = \frac{1}{2}(e^{-\frac{i}{4}\omega t}\,|v_5\rangle - e^{\frac{3i}{4}\omega t}\,|v_7\rangle) - \frac{e^{-\frac{i}{4}\omega t}}{\sqrt{2}}\,|v_2\rangle, \qquad \omega = \frac{g\mu_B a}{\hbar}\,. \quad \text{(S28)}$$

Taking the inner product with the initial state $|S\alpha\rangle$ and then evaluating the square of the norm provides the probability that the radical pair will still be found in that state at a later time instance t:

$$|\langle\psi(t)|S\alpha\rangle|^2 = \frac{10}{16} + \frac{6}{16}\cos(\omega t)\,. \quad \text{(S29)}$$

Note that at time $t = 0$ the probability of finding $|\psi(t)\rangle$ in the initial state is 1, as it should be, but then the probability oscillates over time. This oscillation is the so-called mixing of spin states, where some of the initial singlet state is converted into the triplet states, and later converted back into the initial state. If the initial state was an eigenstate of the Hamiltonian, e.g. if $|\psi(0)\rangle = |v_1\rangle$, then no such oscillations, or mixing, would happen since the solution would be $|\psi(t)\rangle = e^{-\frac{i}{4}\omega t}\,|v_1\rangle$.

The initial state considered here was assuming a specific spin state for the magnetic nucleus, but normally the spin state of the magnetic nuclei is not known initially, as the nuclear spins are in thermal equilibrium - only the spin state of the two unpaired electrons is

known. Thus one needs to consider an ensemble where all possible spin states of the nuclei are present at time $t = 0$, and this cannot readily be done using the Schrödinger equation. This is mainly why the Liouville-von Neumann equation, Eq. (7), is commonly used in spin dynamics, since it can easily account for such mixed spin ensembles.

References

[1] Griffith DJ. Introduction to Quantum Mechanics, 2nd ed. Pearson Prentice Hall; 2005.

[2] Landau LD, Lifshitz EM. Quantum Mechanics: Non-Relativistic Theory. Elsevier Butterworth-Heinemann; 1981.

[3] Sakurai JJ, Napolitano JJ. Modern Quantum Mechanics, 2nd ed. Pearson; 2014.

[4] Merzbacher E. Quantum Mechanics, 3rd ed. Wiley and Sons; 1998.
