# Supplementary material for: Towards predicting intracellular radiofrequency radiation effects
Source: PLoS One. 2019 Mar 14;14(3):e0213286. doi: 10.1371/journal.pone.0213286 (PMC6417702; doi:10.1371/journal.pone.0213286)
Supplement: S2 File — The basic concepts of rotation matrices that describe transformation from the molecular frame of reference to the laboratory frame. (PDF) [file pone.0213286.s003.pdf]

S2 Rotation matrices

The rotation matrix $\mathbf{R}(\Omega)$ used in Eq. (6) of the main paper describes the transformation from the molecular frame of reference to the laboratory frame. The rotation matrix can be constructed in different ways; one way is to use Euler rotation matrices about the x, y and z-axes:

$$\mathbf{R}_x(\theta) = \begin{pmatrix} 1 & 0 & 0 \\ 0 & \cos\theta & -\sin\theta \\ 0 & \sin\theta & \cos\theta \end{pmatrix}, \quad \mathbf{R}_y(\theta) = \begin{pmatrix} \cos\theta & 0 & \sin\theta \\ 0 & 1 & 0 \\ -\sin\theta & 0 & \cos\theta \end{pmatrix}, \tag{S1}$$

$$\mathbf{R}_z(\theta) = \begin{pmatrix} \cos\theta & -\sin\theta & 0 \\ \sin\theta & \cos\theta & 0 \\ 0 & 0 & 1 \end{pmatrix}.$$

Here $\mathbf{R}_x(\theta)$, $\mathbf{R}_y(\theta)$ and $\mathbf{R}_z(\theta)$ performs a rotation of the angle θ about the x-, y- and z-axis, respectively. In the case of an arbitrary three-dimensional rotation, the matrices can be combined to form a new rotation matrix:

$$\mathbf{R}(\alpha, \beta, \gamma) = R_z(\gamma) R_x(\beta) R_z(\alpha), \tag{S2}$$

where α, β and γ are the three Euler angles, defined in S1 Fig. The operator in Eq. (S2) first rotates an angle α about the z-axis, then an angle β about the x-axis, and finally an angle γ about the z-axis again. This is one of the most general forms of three-dimensional rotation that transforms the (x, y, z) coordinate frame into a newly oriented (x', y', z') one. Note that the order of the rotation matrices in $\mathbf{R}(\alpha, \beta, \gamma)$ matters as rotation matrices do not commute. An orientation such as Ω in the main paper corresponds to a set of values for α, β and γ.
